# Development of Coaching Support for LiveWell: A Smartphone-Based Self-Management Intervention for Bipolar Disorder

**DOI:** 10.2196/25810

**Published:** 2021-03-24

**Authors:** Cynthia A Dopke, Alyssa McBride, Pamela Babington, Geneva K Jonathan, Tania Michaels, Chloe Ryan, Jennifer Duffecy, David C Mohr, Evan H Goulding

**Affiliations:** 1 Department of Psychiatry and Behavioral Sciences Feinberg School of Medicine Northwestern University Chicago, IL United States; 2 General Pediatrics Loma Linda Children’s Hospital Loma Linda, CA United States; 3 Department of Social Work UPMC Western Psychiatric Hospital Pittsburgh, PA United States; 4 Department of Psychiatry University of Illinois at Chicago Chicago, IL United States

**Keywords:** human support, adherence, self-management, behavior change, mHealth, bipolar disorder

## Abstract

Despite effective pharmacological treatment, bipolar disorder is a leading cause of disability due to recurrence of episodes, long episode durations, and persistence of interepisode symptoms. While adding psychotherapy to pharmacotherapy improves outcomes, the availability of adjunctive psychotherapy is limited. To extend the accessibility and functionality of psychotherapy for bipolar disorder, we developed LiveWell, a smartphone-based self-management intervention. Unfortunately, many mental health technology interventions suffer from high attrition rates, with users rapidly failing to maintain engagement with the intervention technology. Human support reduces this commonly observed engagement problem but does not consistently improve clinical and recovery outcomes. To facilitate ongoing efforts to develop human support for digital mental health technologies, this paper describes the design decisions, theoretical framework, content, mode, timing of delivery, and the training and supervision for coaching support of the LiveWell technology. This support includes clearly defined and structured roles that aim to encourage the use of the technology, self-management strategies, and communication with care providers. A clear division of labor is established between the coaching support roles and the intervention technology to allow lay personnel to serve as coaches and thereby maximize accessibility to the LiveWell intervention.

## Background

Bipolar disorder is a serious mental illness characterized by recurrent episodes of mania, hypomania, depression, and mixed states [1,2]. Even with pharmacological treatment, recurrence of acute episodes, long episode durations, and persistence of interepisode symptoms leads to significant disability, with three-quarters of those affected never achieving full recovery of psychosocial function [3-8]. The addition of empirically supported psychotherapy to pharmacotherapy improves clinical and recovery outcomes [4,9], but only half of individuals with bipolar disorder receive any therapy [10,11].

Mental health technologies (MHTs), including web and smartphone-based applications, provide a means to increase the availability of empirically supported psychotherapeutic strategies for managing mental health problems. Additionally, relative to face-to-face (F2F) treatment, MHTs can increase the functionality of interventions by providing real-time assessments, feedback, and provider alerts. MHTs have been developed and found to be effective for depression and anxiety, and recent efforts have begun to focus on serious mental illnesses such as bipolar disorder and psychosis [12-17].

To extend the accessibility and functionality of psychotherapeutic strategies for bipolar disorder, we developed LiveWell, a smartphone-based self-management intervention. Unfortunately, many MHTs suffer from high attrition rates where users rapidly fail to maintain their use of the technology [18,19]. To address this challenge, human support has been introduced to help individuals utilize MHTs. This support often includes technical support to ensure that the technology is working as intended and use support to ensure that the technology is being used. It may also include clinical support to ensure that users identify the content and tools relevant to their needs, use them correctly, and translate this use into their daily lives [20]. Providing technical and use support has been shown to reduce attrition and improve adherence with MHTs, but this increased engagement is not always accompanied by improved outcomes [18,20-24]. Including clinical support may result in improved outcomes [20]. However, limited description of what this clinical support involves has made it difficult to replicate, improve, and implement this type of human support for MHTs [20,25-27].

To facilitate ongoing efforts to develop human support for MHTs, this paper describes the design decisions, theoretical framework, content, mode, and timing of delivery, as well as the training and supervision for coaching support, of the LiveWell technology. The design and development of LiveWell was guided by an intervention mapping and person-centered approach utilizing an iterative strategy (G. Jonathan et al, unpublished manuscript, 2021) [28-33]. This included an initial field trial of a simple self-monitoring application followed by the development of the complete self-management intervention (NCT02405117) via design interviews, usability testing, and a pilot trial. The final intervention design is being tested in a randomized control trial (NCT03088462). The supportive accountability model, which focuses on application use support, served as the starting point for the coaching development process (G. Jonathan et al, unpublished manuscript, 2021). However, F2F therapies for bipolar disorder involve high levels of personalization to meet individuals’ varying clinical needs, symptom states, and commitment to self-management. As such, additional support roles were created to better meet the needs of people with bipolar disorder and potentially improve both adherence and outcomes. Specifically, 3 structured coach roles were developed to support (1) application use, (2) self-management, and (3) communication with clinical care providers.

## Division of Labor

**Figure 1:**
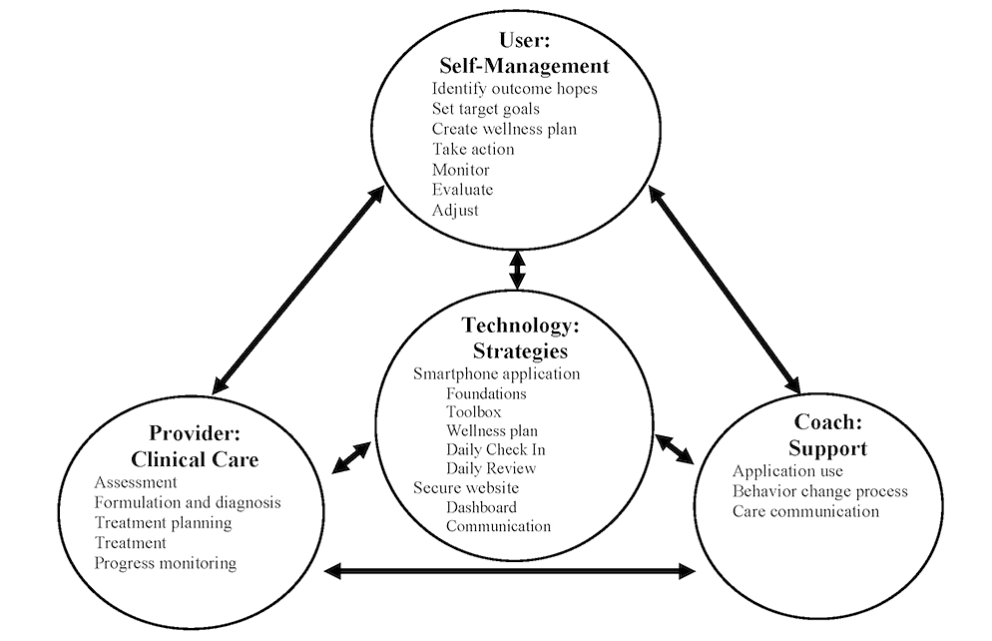
LiveWell division of labor.

To reduce costs and increase access, human support for MHTs often involves personnel without professional training in mental health care [34-36]. To facilitate the use of lay personnel, the LiveWell coach’s responsibilities are clearly defined in relation to the user, provider, and technology (Figure 1). The coach does not serve as a therapist but instead facilitates the use of the technology. Empirically supported psychotherapeutic strategies, such as providing information and assessments, goal setting, planning, monitoring, and suggesting skills to practice and adjustments to make, are embedded in the LiveWell technology. To ensure the coach operates within the scope of nonclinical practice, there is a clear division of labor between the technology and the coach. The technology operates as the psychotherapeutic strategy expert and provides status summaries and alerts to the coach who uses flowsheets and structured scripts to serve as a technology use concierge.

## User and Provider

LiveWell is an adjunctive intervention and requires users to be actively engaged with a psychiatrist. The psychiatrist is expected to work with the user to come to a mutual understanding of clinical problems and treatment plans and engage in active and sustained collaborative treatment and progress monitoring. Consistent with this chronic disease self-management model of care [37-48], the LiveWell intervention aims to support the user in learning and utilizing appropriate self-management strategies, including effective communication with their provider. The intervention also aims to assist the provider by delivering clinical information and alerts based on real-time user assessments. Overall, LiveWell seeks to support the functioning of the user-provider dyad to reduce problems caused by bipolar disorder and support the achievement of life goals.

## Technology

The LiveWell technology supports the user in learning about and engaging in empirically supported self-management strategies through the delivery of information and assessments, provision of goal setting, planning, and monitoring tools, as well as dynamically suggesting materials to review, skills to practice, and adjustments to make (Figure 1). It presents foundational information on bipolar disorder and self-management (Foundations), a toolbox with a variety of self-assessment surveys and skills instructions (Toolbox), and enables the development of a personalized plan for living well (Wellness Plan). The core of the intervention is a Daily Check-In, where users are asked to monitor medication adherence, sleep duration, routine, and wellness ratings. Based on data from the Daily Check-In, an expert system (Daily Review) provides interactive, personalized feedback and directs individuals to relevant aspects of the application. Users also complete a Weekly Check-In of depressive and manic symptoms: the 8-item Patient Health Questionnaire (PHQ-8) and Altman Self-Rating Mania Scale (ASRM) [49,50]. Users, providers, and coaches can access a secure website that contains a summary of the self-assessment data. The expert system also provides email alerts to providers and coaches when clinical support may be needed.

## Coach

The LiveWell coach supports application use adherence, self-management, and clinical care communication. The technology provides data summaries and alerts that direct the coach toward areas to be addressed. All coach-user conversations utilize structured scripts and protocols, which were refined based on user and coach feedback. Coaching starts with a F2F meeting that addresses how LiveWell can help the user utilize self-management strategies to live well with bipolar disorder (Multimedia Appendix 1, Application Training Script). Coaches then complete scheduled calls with users to encourage application use and support self-management (Multimedia Appendix 1, Scheduled Call Scripts). In addition, coaches may be prompted by the technology to contact users regarding application use adherence problems or to facilitate communication with providers when users’ self-assessments indicate issues with treatment adherence, the presence of early warning signs, or worsening or severe symptoms (Multimedia Appendix 1, Ad Hoc Calls Script).

## Application Use

### Rationale

Systematic reviews of MHTs demonstrate that technical and application use support improves adherence and decreases attrition [51-53]. To develop this support role for LiveWell, we used the supportive accountability model, which is effective and proposes that accountability to a coach perceived as trustworthy, benevolent, and knowledgeable enhances adherence to MHTs (G. Jonathan et al, unpublished manuscript, 2021) [54]. To build accountability, the coach collaborates with the user to form clear, process-oriented expectations regarding how the use of the technology might help them achieve intrinsically motivated hopes related to the desired intervention outcomes, including decreasing relapse risk and interepisode symptoms as well as improving quality of life.

### Implementation

The initial F2F coach meeting (60-75 minutes long) aims to establish a relationship with the user in which the coach is viewed as a trustworthy, benevolent, and knowledgeable support. The meeting starts with introductions followed by elicitation of the user’s hopes (“What would you like to be different at the end of this program?”). The idea that the LiveWell technology can aid in using self-management strategies to stay well is introduced and discussed. The meeting’s focus then shifts to support the user in engaging with the technology to learn about and utilize self-management strategies. The F2F meeting ends with explaining the coach’s roles, including a clear summary of expectations regarding application use and when the coach might contact the user about use adherence or clinical care concerns. The coach ends the meeting by encouraging commitment to application use and acceptance of adherence monitoring to reinforce accountability.

**Figure 2:**
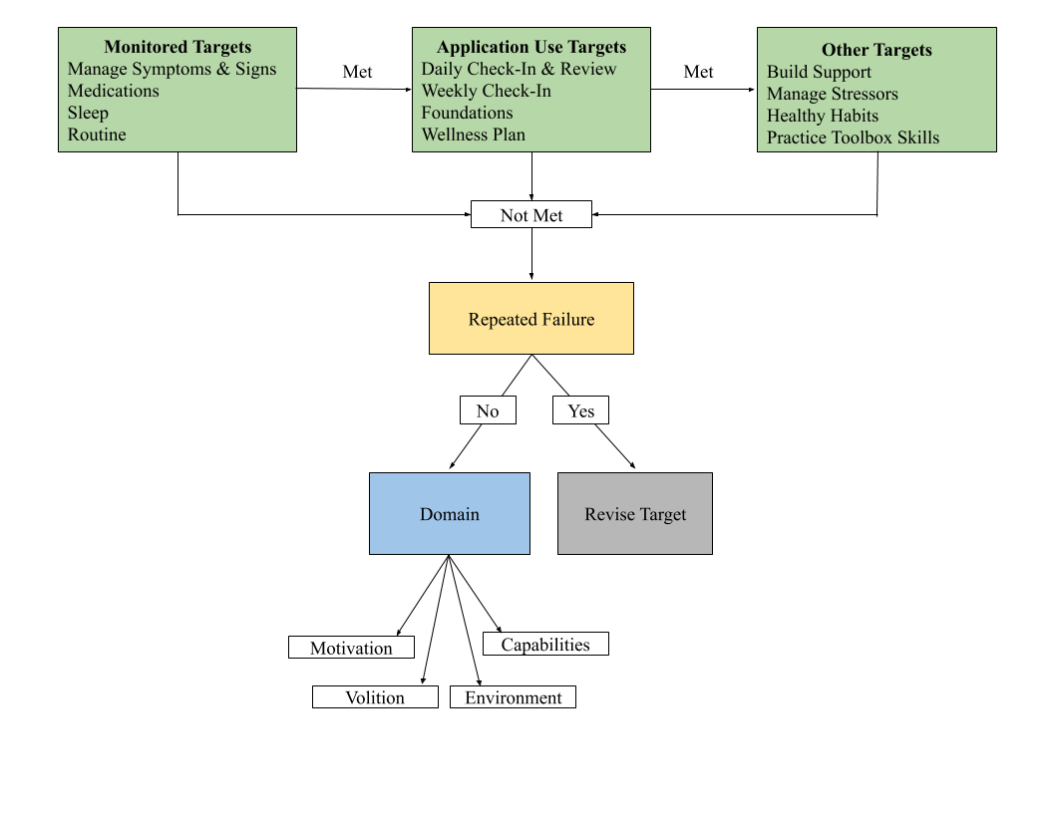
Scheduled coach calls.

During scheduled follow-up calls, the coach summarizes application use adherence since the previous call (Daily and Weekly Check-Ins completed; Foundation lessons, Daily Reviews and Toolbox content viewed). The coach then supports adherence and explores barriers to use when nonadherence occurs (Figure 2). The coach may also contact users (via calls, text messages, or emails) when the technology delivers nonadherence alerts to the coach. These alerts are triggered by the user missing Daily Check-Ins (≥ 3 times in a week) or Weekly Check-Ins (≥ 2 weeks in a row). During these ad hoc contacts, coaches elicit barriers to use and direct the user to sections of the application that may help them overcome these barriers. Coaches also provide ad hoc technical support to update application content and manage general technical issues.

## Self-Management

### Rationale

Recent randomized trials suggest that MHT human support focused on technical and application use support does not consistently improve clinical outcomes [18,20-24]. Therefore, coach roles may need to be expanded to include clinical support. For example, the efficiency model of support suggests additional assistance is necessary for psychoeducation and implementation of skills, as well as alignment of these skills with users’ unique needs [20]. Users with bipolar disorder may also specifically benefit from guidance in navigating self-management challenges [55]. To develop self-management support for LiveWell, we integrated information from empirically supported psychotherapies for bipolar disorder, the health psychology behavior change literature, and chronic disease self-management models [25,26,30-32,37,38,40,41,46-48,56-64].

LiveWell guides users to address specific behavioral targets, including medication adherence, obtaining adequate sleep duration, and maintaining regular routines. LiveWell emphasizes the importance of identifying early warning signs and symptoms of relapse, as well as developing and implementing a plan for managing these signs and symptoms. In addition, strengthening social support, managing stressors, and healthy habits regarding diet, exercise, and substance use are also addressed. These targets were selected because they have been proposed to underlie the improved outcomes (ie, reduced episode recurrence and interepisode symptoms, and improved quality of life) produced by empirically supported therapies for bipolar disorder [8,57,65].

To facilitate changes in these behavioral targets, the technology and coach deliver behavior change techniques (BCTs) that constitute the smallest intervention components impacting behavioral regulation [25,26,30-32,58-62]. BCTs can be grouped into nonoverlapping clusters hypothesized to alter specific behavioral determinants involved in enacting target behaviors [61,62,66-69]. Determinants and their corresponding techniques can be grouped into 4 domains: motivational determinants involved in developing an intention to engage in a behavior, volitional determinants involved in enacting the behavior, and environmental determinants and capabilities that impact both motivational and volitional processes. When difficulties arise for users in achieving their target goals, attention to these domains allows coaches to guide users to application content that addresses barriers and suggests solutions to reach their target goals.

The coach provides self-management support through behavior change counseling, a simplified adaptation of motivational interviewing that is effective with brief consultations administered by individuals without professional training in mental health care [70,71]. The goal is to encourage users to express commitment to and confidence in developing, enacting, and adjusting their desired behavior change plans. The coach provides support for setting appropriate target goals, personalizing a wellness rating scale and plan, and monitoring progress. When target goals are not met, the coach offers application content guidance to explore barriers and find solutions. When target goals are met, the coach reinforces target success and links this success to outcome hopes.

### Implementation

At the F2F meeting, the coach works with the user to review their experiences with normal ups and downs, early warning signs and symptoms, episodes, and crisis situations. This review leads to the development of a personalized 9-point wellness rating scale to facilitate self-monitoring. The coach walks the user through the application and has the user complete a Daily Check-In and Daily Review. As part of this practice, the user sets specific behavioral targets for medication adherence, sleep duration, routine bedtime and rise time, and wellness rating range. The coach encourages the user to set parameters known to facilitate health, such as aiming to take their medications 100% of the time, sleep the recommended amount each day (7 to 9 hours, or 6 to 10 hours acceptable for some), go to bed and start their day within a 1.5-hour window, and keep their wellness ratings within a “balanced” range (with expected ups and downs due to routine events) [8,57,65,72].

Six scheduled coaching calls occur during weeks 1-4, 6, and 16. Each call lasts about 15 minutes, except for a longer week-4 call (~30 minutes), during which the Wellness Plan is personalized. Before each call, the coach reviews a dashboard that summarizes the user’s application use and the percent of days their personalized target goals were met. The coach uses this summary and a flowsheet (Figure 2) in conjunction with call scripts to guide discussion and balance user autonomy needs with target priorities.

Each call starts with agenda-setting and invites collaboration. Next, the coach and user review progress toward the user’s personalized target goals. If users fail to meet their personalized goals, the flowsheet and scripts provide tips to help the coach focus on behavior change domains relevant to their current situation. The coach encourages gentle exploration, aware that motivational, volitional, environmental, or capability determinants may have hindered progress. A tip sheet directs coaches to application sections relevant to users’ specific behavioral target goals and behavior change determinants (Multimedia Appendix 1). If the report indicates that the user successfully met their target goals, the coach provides reinforcement and asks how this success might help achieve their outcome hopes. If application use adherence was not maintained, this is addressed. Otherwise, the coach works with the user to identify other personalized goals for using the application, such as building supports, managing stressors, creating or maintaining healthy habits, and practicing toolbox skills (Figure 2).

During the week-4 call, the coach helps the user personalize their wellness plan by guiding them to application content relevant to developing task and coping plans for each behavioral target. The user is also given an opportunity to make changes to their wellness rating scale and adjust their target goals. Going forward, the coach encourages the user to use the Wellness Plan to reduce risk when feeling well and take action when experiencing symptoms. At the end of each call, based on target progress during the prior week and the user’s priorities, the user and the coach collaboratively set a target goal for the week ahead. Users are urged to link their target behavior goals with their initial outcome hopes and are also asked to anticipate any potential obstacles and ways to overcome them. To assess users’ experience of the psychoeducational materials, the coach also asks users if they had any thoughts, feelings, or questions about the materials. Each call ends with a recap of the personalized target goals for the week ahead. In summary, the coach acts as a concierge; directed by a simplified data summary, a flowsheet, and structured scripts, the coach suggests appropriate application content and tools to match users’ needs in addressing their target goals.

To provide a detailed view of the BCTs that may be delivered by the coach, the F2F application training and scheduled call scripts were coded using published protocols for coding BCTs [62,73,74]. This coding allows the percent of pages (PP) with content addressing specific outcomes, targets, determinants, domains, and BCTs to be quantified (Multimedia Appendix 2). In addition, the BCTs were ranked (Rank) based on their coding frequency; the top 15 ranked BCTs are displayed in Table 1. In terms of outcomes, symptoms and episodes were most commonly addressed in the coaching script content (69%). In terms of targets, over half of the coaching script content addressed managing symptoms and signs and building supports (63%). In terms of determinants, support, planning, self-efficacy, intention, attitudes, and perceptions constituted the bulk of the content (70%), with agenda mapping, emphasize autonomy, coping planning, open-ended and desire-ability-reason-need questions, and review behavior goals being the top 5 ranked BCTs (42%).

**Table 1:** LiveWell coaching script content.

Domain (PP^a^), determinant (PP), and technique (PP)			Rank^b^
**Motivation (39.4)**			
	**Self-efficacy (12.3)**		
		Emphasize autonomy (8.5)	2
		Affirmation (3.8)	8
	**Intention (8.9)**		
		Agenda mapping (8.9)	1
	**Attitudes & perceptions (9.2)**		
		Desire-ability-reason-need questions (4.8)	5
		Elicit-provide-elicit (2.4)	12
		Information about health consequences (2.0)	14
	**Insight (4.5)**		
		Guided discovery (4.5)	6
	**Knowledge (4.5)**		
		Information about a health condition (4.5)	6
**Volition (25.9)**			
	**Planning (13.3)**		
		Coping planning (7.5)	3
		Task planning (3.8)	8
		Consider change options (2.0)	14
	**Adjustment (4.8)**		
		Review behavior goals (4.8)	5
	**Goal setting (2.9)**		
		Process goal (2.9)	10
	**Evaluation (2.6)**		
		Feedback on behavior (2.6)	11
	**Monitoring (2.3)**		
		Self-monitoring of behavior (2.3)	13
**Environment (24.9)**			
	**Support (21.1)**		
		Open-ended questions (7.2)	4
		Permission to provide information and advice (4.4)	7
		Social support (practical; 3.8)	8
		Social support (unspecified; 3.4)	9
		Support change/persistence (2.3)	13
	**Prompts (2.0)**		
		Introduce cues (2.0)	14
	**Reinforcement (1.8)**		
		Social reward (1.8)	15

^a^PP: percent of total coaching script pages.

^b^Rank: behavioral change techniques rank-ordered based on their PP, BCTs with same PP given same rank-order.

## Care Linkage

### Rationale

Timeliness of care, especially during the onset of early warning signs, can significantly impact the course of a manic or depressive episode [75]. Additionally, increased communication and collaboration with a care provider are associated with reduced suicide risk, better medication adherence, and greater satisfaction with care [76,77]. For individuals with bipolar disorder, research shows that the use of a chronic disease self-management model reduces the number of weeks spent in manic episodes and improves overall functioning [78,79]. The LiveWell intervention strives to increase early intervention of illness-related problems by enhancing client-provider communication, a common target of empirically supported psychotherapy for bipolar disorder [9,80,81]. We structured the coach care linkage role using the chronic disease self-management model, wherein the coach utilizes active and sustained follow-up to monitor users’ status, identify problems, and reinforce progress in implementing the care plan [81,82].

### Implementation

LiveWell aims to enhance user-provider communication by prompting users to seek help when necessary and activating providers by offering access to a secure, web-based clinical status summary portal and automated email alerts. Within this system, the coach provides support to both users and providers. When a user experiences poor medication adherence, significant sleep duration changes, or the onset of early warning signs or worsening symptoms, the coach receives an automated email alert. Enrolled providers also receive an automated email or a phone call from the coach based on the provider’s preference. When calling providers, the coach summarizes recent check-in data, reminds the provider that a self-assessment data summary is available on the secure portal, and welcomes the provider to reach out with questions.

**Figure 3:**
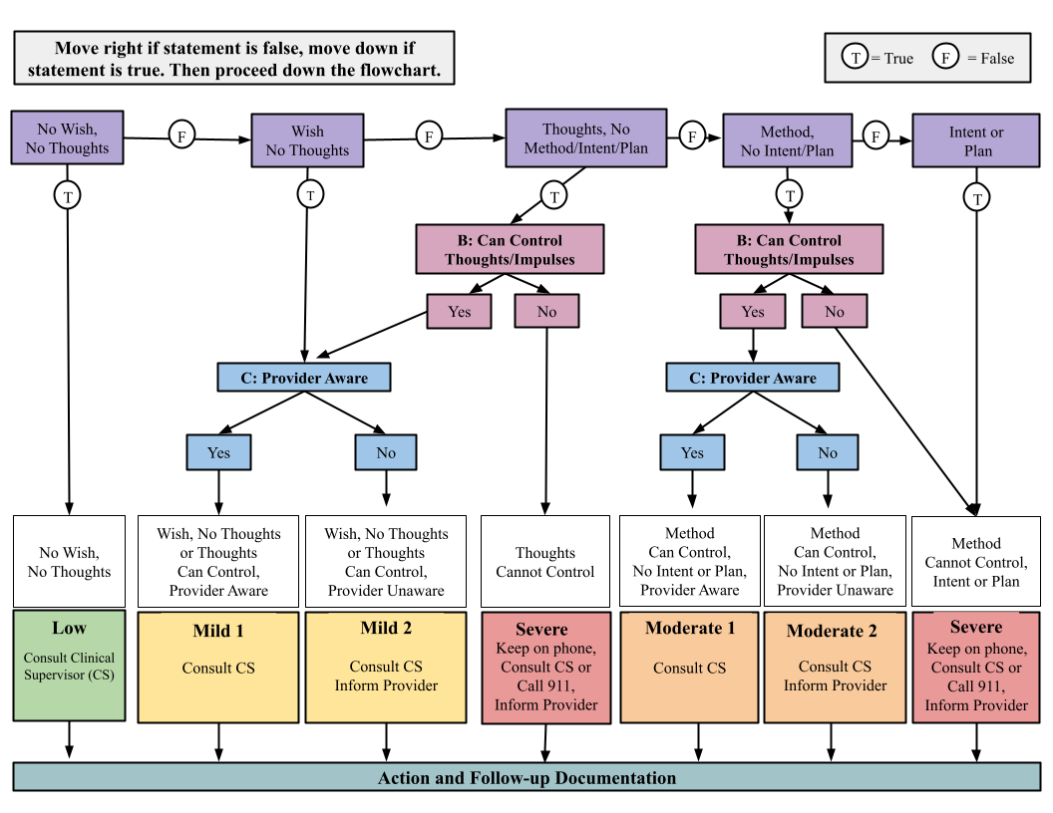
Suicidality assessment protocol flowsheet. CS: clinical supervisor.

**Figure 4:**
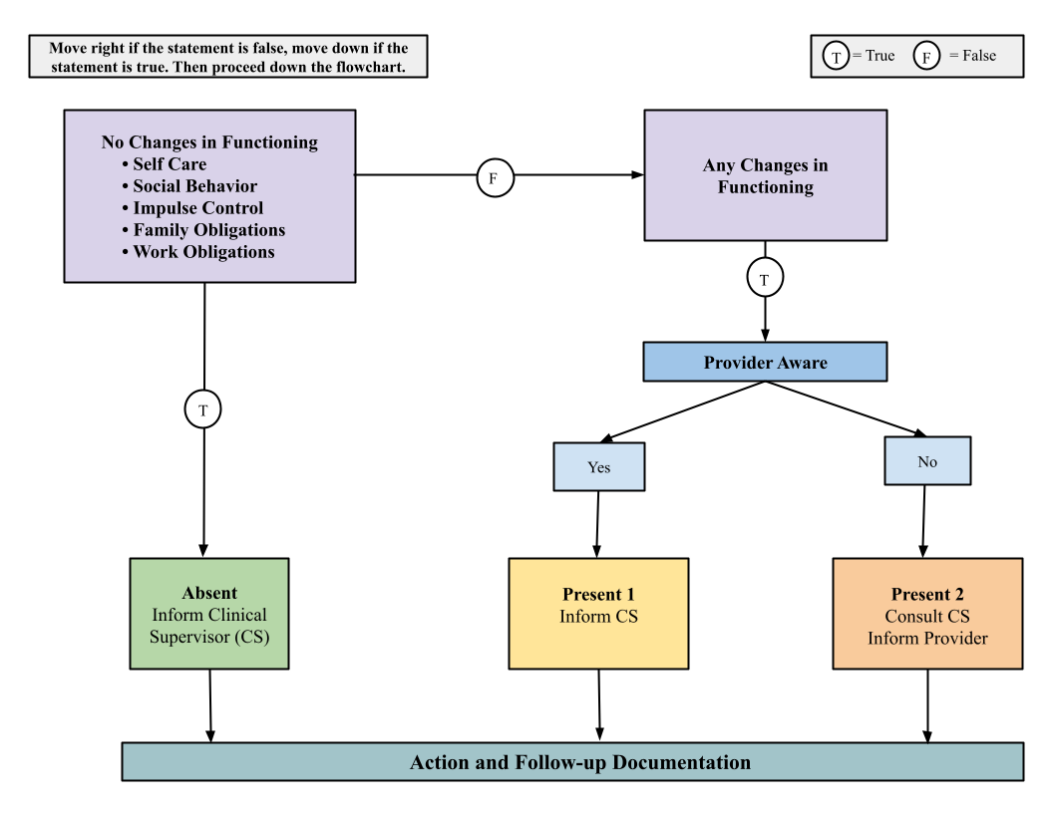
Functional evaluation protocol flowsheet. CS: clinical supervisor.

Additionally, if a user enters a crisis rating on the Daily Check-In (+4 or -4) or has a Weekly Check-In score consistent with the onset of a mood episode (ie, a change from a below- to an above-threshold score: a PHQ-8 score of ≥ 9 or an ASRM score of ≥ 6), the coach receives an email alert [49,50]. In this case, the coach performs a suicidality assessment and functional impairment evaluation (SAFE). The coach utilizes a structured protocol (Multimedia Appendix 1) and flowsheets (Figures 3-4) to guide this risk evaluation and follow-up. After the alert is received, the coach attempts to contact the user up to 3 times within 24 hours to complete the SAFE protocol. If the coach cannot reach the user, the coach reaches out to the user’s psychiatrist with available clinical information provided by the user’s self-assessments.

The suicidality assessment (SA) protocol contains 3 sections: assessing suicidal ideation, the ability to control these thoughts, and the providers’ awareness of the information provided. The assessment of suicidal ideation utilizes the Columbia Suicide Severity Rating Scale (C-SSRS) [83]. Once the coach has reached the user by phone, the coach explains that they are calling due to the alert. The coach then explains that they are following up based on the safety monitoring discussed at the F2F meeting. The coach starts by asking 2 questions from the C-SSRS [83]: “Have you wished you were dead or wished you could go to sleep and not wake up?” (Wish); “Have you actually had any thoughts about killing yourself?” (Thoughts). Based on the user’s response to these 2 questions, the coach then follows the logic of the SA protocol and flowsheet (Figure 3) to determine the user’s risk level (low to severe risk), the provider’s awareness, and the actions that the coach needs to take. This protocol allows coaches to proactively implement a standardized suicidal ideation evaluation, efficiently communicate the information they obtain, and promptly receive clinician support.

The functional impairment evaluation (FE), which draws upon components of the World Health Organization Quality of Life Questionnaire, Lehman’s Quality of Life Interview, and the Global Assessment of Functioning [84-86], assesses users’ life circumstances to evaluate impairment in daily activities. The user is asked if there is any new onset of changes or problems in their self-care, social activities, impulse control, or meeting of family or work obligations. If so, the coach asks if their provider is aware of this information and follows the logic of the FE protocol and flowsheet (Figure 4) to determine the actions to take. This procedure ensures that the coach is not responsible for determining the clinical significance of the reported changes in functioning.

## Training and Supervision

Coaches receive initial training and weekly supervision. Prior to a full-day workshop, they are given a training manual (Multimedia Appendix 1), coaching scripts (Multimedia Appendix 1), and several reference papers. The training manual includes information about bipolar disorder and treatment, the LiveWell program, supportive accountability, behavior change processes, and coaching skills and responsibilities. The core training occurs in one full-day workshop. The morning involves a review and discussion of the material presented in the training manual. Case examples are provided to elucidate the material. The afternoon involves learning about the core coaching skills. All coaches engage in role-playing of scheduled calls with a supervising clinical psychologist. They train to mastery, defined as 3 consecutive contacts evaluated at or above 85% satisfactory ratings on an adapted version of the behavior change counseling index (Multimedia Appendix 1) [87]. Once some trained coaches are available, new coaches also sit in on experienced coaches’ initial visits and coaching calls. Experienced coaches then observe the new coaches’ interactions to provide direct feedback. Beyond the initial training, weekly group supervision is provided. Supervision includes addressing any questions that arise during the week and feedback on audiotapes of coach F2F training or calls randomly selected for review.

Coaches also receive additional crisis support training. Supervisors and experienced coaches meet with new coaches and review the SAFE protocol and situations in which the protocol is activated. Coaches listen to 6 selected audio-recorded calls covering different risk levels during which the protocol was activated and successfully administered. They also shadow 3 live crisis calls and complete 3 calls under the supervision of an experienced coach. The training concludes with a protocol quiz regarding risk assessment in sample scenarios (Multimedia Appendix 1). New coaches are required to receive a score of 100%.

Themes and subthemes from the supervision of coaches (*N*=7) trained during our recent studies identify the importance of teaching lay personnel how to conduct structured interactions with users. As outlined in Table 2, posttraining supervision needs fell into 3 categories: (1) general counseling, (2) behavior change processes, and (3) LiveWell content. In the area of general counseling, coaches need additional instruction on when and how to point users back to parts of the application, how to respond to negative life events in an empathic way without leading to a deepening exploration of the issue, and how to delicately manage times when the perceptions of the user and coach are at odds. In regard to behavior change processes, coaches also receive further guidance around differentiating overall hopes for LiveWell and specific behavioral targets, how to set target goals as well as task and coping plans, and how to demonstrate understanding and reinforce change. Early on in training, coaches often try to directly engage users in problem-solving before assessing the obstacles involved; supervision engages users in thinking through difficulties in a more systematic and guided way. Supervision also helps coaches respond when users are unable or unmotivated to work on a specific behavioral target. Further, regarding the LiveWell content, coaches and the supervisor agree that an initial training followed by staged training is most effective. Staged training supervision sessions focus on Daily Check-Ins, the Wellness Plans, Foundation lessons, and the Toolbox. It appears that within 3 months, routine supervision feedback for new coaches is expended, and they can navigate encounters with mastery.

**Table 2:** Supervision themes and subthemes.

Themes and subthemes	
**General counseling**	
	Maintaining integrity of coaching role
	Establishing and maintaining boundaries
	Responding to reports of negative life events
	Managing discrepant coach-participant perceptions
**Hows and whys of behavior change**	
	Differentiating between hopes and targets
	Setting goals, task, and coping plans
	Understanding and reinforcing change
	Assessing challenges before problem-solving
	Reacting to inability or amotivation to work on specific targets
**LiveWell content**	
	Staged training
	Suggestions guide
	Daily Check-in
	Wellness Plan
	Foundations
	Toolbox

## Discussion

This paper describes the design decisions, theoretical framework, content, mode, timing of delivery, and the training and supervision for coaching support of the LiveWell technology. The development of this support initially focused on using the supportive accountability model to facilitate technology use adherence and reduce attrition. However, during the expansion of the application from a simple self-monitoring tool to a complete self-management application, feedback from users and coaches suggested that the intervention might benefit from expanding the coaching roles. Thus, to support the clinical needs of individuals with bipolar disorder and potentially improve outcomes, coaching was expanded to include supporting self-management and communication with care providers.

To develop the LiveWell coaching support, we combined information from empirically supported psychotherapies for bipolar disorder, the health psychology behavior change literature, and chronic disease self-management models. In addition to guiding the development of the clinical support roles, this integration provides the ability to label, measure, and relate changes in (1) target behaviors proposed to improve outcomes, (2) behavioral determinants proposed to govern enactment of target behaviors, and (3) exposure to behavior change technique content and tool use proposed to alter behavioral determinants. This integrated approach to developing the coaching roles was initially used to develop the smartphone application content and derived from using an intervention mapping approach. Hopefully, using the same process to develop and investigate the mechanisms of action of the technology and its coaching support will provide a generally applicable means of further understanding how supported MHTs work and clarify how best to balance intervention delivery via technology and human support.

In developing the coaching support for LiveWell, we aimed to define a clear division of labor between the coaching roles and the technology to facilitate the use of personnel without professional training in mental health care. The use of a chronic disease self-management model was useful in defining the coaching support tasks in relation to the technology, the user, and the provider. To assist the coach, reports summarizing progress on clearly defined and monitored behavioral target goals and clinical alerts are provided by the technology. This information, combined with structured scripts, protocols, and flowsheets, allows the coach to deliver clinical support without extensive mental health training. Consequently, this should improve accessibility, as lay personnel can reduce costs and increase access to MHTs.

Obtaining feedback from users and coaches played an important role in developing the division of labor between the technology and coaching support. To facilitate coaches’ use of the reports and alerts, users and coaches required a clear understanding of the behavioral target goals and the rationale for the clinical alerts. As a result, user and coach feedback led to multiple iterative revisions of the technology content and the coaching reports and alerts to ensure that the coaches felt confident in acting on the information provided by the technology. Developing straightforward, structured scripts and protocols for delivering self-management support and communicating with care providers was also essential in allowing the coaches to carry out these roles. In addition, the use of flowsheets appeared to assist lay personnel with learning the protocols.

The development of clear flowsheets and protocols was especially significant in allowing lay personnel to deliver the suicidality assessment and functional evaluation without undue distress. It is important to note that the use of lay personnel in this assessment role requires the availability of a trained clinical professional to provide real-time support to the coaches when severe suicidal ideation is detected. This need for real-time professional clinical support for the coaches and a mental health professional to deliver the coach training requires careful consideration when planning for implementing and disseminating supported MHT interventions. This consideration is especially relevant when these interventions are directed toward high-risk populations such as individuals with bipolar disorder.

MHT coaching is a nascent field. As research develops, we anticipate that the coaches’ effectiveness at improving adherence and clinical outcomes produced by MHT interventions will increase. During the design and development of the LiveWell intervention, using the same framework to develop the technology’s content and tools and its coaching support played an important role in enabling a clear division of labor to be defined between the technology and the coach. In addition, obtaining feedback from both users and coaches was critical in developing both the technology and the coaching support and assisting coaches without professional mental health training to learn and deliver the coach roles. We hope that providing a detailed explanation of the rationale and implementation of our coaching support roles for LiveWell will contribute to the development of more effective coaching support for MHT interventions.

## Appendix

Multimedia Appendix 1 LiveWell coaching materials.

Multimedia Appendix 2 Coding breakdown of the coaching scripts.

## Abbreviations

ASRM: Altman Self-Rating Mania Scale
BCT: behavior change technique
C-SSRS: Columbia Suicide Severity Rating Scale
FE: functional impairment evaluation
F2F: face-to-face
MHT: Mental health technology
PHQ-8: 8-item Patient Health Questionnaire
PP: percent of pages
SA: suicidality assessment
SAFE: suicidality assessment and functional impairment evaluation
