# Supplementary material for: Development of Coaching Support for LiveWell: A Smartphone-Based Self-Management Intervention for Bipolar Disorder
Source: JMIR Form Res. 2021 Mar 24;5(3):e25810. doi: 10.2196/25810 (PMC8075075; doi:10.2196/25810)
Supplement: Multimedia Appendix 1 [file formative_v5i3e25810_app1.pdf]

LiveWell Coaching Materials

I. Training Manual

II. Application Training Script

III. Scheduled Call Scripts

IV. Scheduled Call Script Tips

V. Ad Hoc Calls Script

VI. Suicidality Assessment Protocol

VII. Suicidality Assessment Protocol Quiz

VIII. Functional Evaluation Protocol

IX. Fidelity Rating Scale

I. Training Manual

LiveWell Program

Coach Training Manual

Cynthia A. Dopke, Ph.D.
Evan Goulding, M.D., Ph.D.

© Northwestern University

PREFACE

Welcome to the *LiveWell* program! We are glad to have you as part of the team. As a coach, you play a critical role assisting individuals in managing their bipolar disorder and clinicians in providing more timely care. You will offer support to participants as they learn to use a smartphone application designed to help them manage their bipolar disorder. Given your contact with participants, you will also provide feedback to their clinicians about how things are going with their participants. This manual offers guidance for you in your role as a coach. It describes the context in which you will be operating, and it offers direction for your specific duties. It provides an overview of bipolar disorder, the state-of-the-science treatments for bipolar disorder, and our protocol designed to advance the treatment of bipolar disorder. It also describes in some detail the basic competencies you will rely upon in your work with participants. Based on a model called "supportive accountability" you will engage and motivate those enrolled in the study as well as assist them in the utilization of the application.

This manual is a starting point for your training. It also serves as a reference guide for you down the road. In addition to the materials included here, you will get face-to-face instruction, watch others, and role-play with feedback all prior to starting as a coach. So read, wonder, and ask questions as you go through your training!

Cynthia A. Dopke, Ph.D.
Assistant Professor of Psychiatry and Behavioral Sciences
Feinberg School of Medicine, Northwestern University

Evan Goulding, M.D., Ph.D.
Assistant Professor of Psychiatry and Behavioral Sciences
Feinberg School of Medicine, Northwestern University

TABLE OF CONTENTS

PREFACE 1
INTRODUCTION 3
TRAINING SCHEDULE 5
BASIC FACTS ABOUT BIPOLAR DISORDER 6
TREATMENTS FOR BIPOLAR DISORDER 12
LIVEWELL PROGRAM 15
SUPPORTIVE ACCOUNTABILITY 19
BEHAVIOR CHANGE 23
COACHING SKILLS 30
COACHING RESPONSIBILITIES 35
WRAPPING UP 37
REFERENCES 38

1

INTRODUCTION

The *LiveWell* program uses a mobile smartphone application to help individuals with bipolar disorder reduce symptoms and full-blown recurrences of their illness. While relying on well-tested therapeutic strategies it is innovative in that it translates current treatments to a self-management format and in particular to a mobile application. While the application is a central part of the program, the coach plays an integral role that is critical to the success of the *LiveWell* program.

Background

There is very good evidence that current pharmacological treatments reduce symptoms and relapse in bipolar disorder. However, multiple acute episodes, long episode durations, and significant inter-episode sub-syndromal symptoms are common in individuals with bipolar disorder even with pharmacological treatment. As a result, individuals are symptomatically ill about half of the time. Recent advances in psychosocial therapies have been developed to improve medication adherence, sleep duration, maintenance of regular daily routines, recognition of early warning signs of illness, and use of relapse prevention plans. Although these psychosocial treatments are effective in controlled clinical trials (e.g. reduced relapse rates, decreased symptom burden, increased quality of life), at most, only half of individuals with bipolar disorder receive any psychosocial treatment. Even fewer receive evidence-based psychosocial treatments from clinicians specifically trained to deliver interventions as delivered in controlled trials. Improving access to and long-term engagement in psychosocial interventions therefore provides an important means to substantially improve bipolar disorder treatment.

In addition, current tools for assisting participants in taking medications, optimizing sleep, and regularizing daily rhythms, as well as monitoring, recognizing, and managing early warning signs of impending acute episodes can be improved. Current strategies rely on subjective participant report at office visits and many individuals have difficulties identifying early warning signs. No current monitoring tools allow daily self-report and direct measurement of observable daily patterns of behavior to be incorporated into routine monitoring and in the moment daily feedback.

The content of *LiveWell* is primarily based on cognitive-behavioral therapies for bipolar disorder, empirically supported treatments offered as an adjunct to medications. Key elements from these therapist-delivered interventions are distilled and translated into a 16-week, self-management program for individuals with bipolar disorder.

Another goal of *LiveWell* is to utilize smartphones to automate collection of self-report and behavioral data including: 1) medication adherence, bedtime/rise time, daily and weekly wellness, 2) standard activity data

which has previously been demonstrated in short-term studies to characterize bipolar participant health status and 3) novel data such as location and social interaction which we anticipate will have substantial utility in monitoring participant status. This data will be incorporated into phone-based tools to enrich participant self-monitoring, provide real-time feedback, and improve participant engagement in disease self-management. Automated processing algorithms also provides clinicians with timely access to easily utilizable data to allow better evaluation and targeting of treatment. The system will be embedded within a care model that emphasizes participant self-management, increased participant-provider communication, and better informed provider intervention. To enhance acceptance, participation, and long-term system use by participants and providers, an ongoing process of iterative, user-centered design will be used for ongoing improvement of the *LiveWell* system.

Your Role

The effectiveness of behavioral intervention technologies (BITs)—applications on computers, smartphones, and tablets—to enhance health behaviors is improved by human support. That is, rather than individuals just using a device on their own, offering a person to help engage, motivate, and utilize programs results in better health outcomes. Support persons act not as clinicians, but rather as coaches. Behavioral intervention technologies are self-contained in that clinical information is covered by the program, and individuals using these programs are directed to contact their clinicians when further clinical intervention is necessary.

Your role will be that of a support person, which we refer to as a coach. You will guide and direct participants in our study as they learn to use the *LiveWell* application. Like a coach, you will be on the sidelines directing and advising. The goal is for you to help increase their adherence in using the application most effectively. You will engage, encourage, and problem solve with the participants.

This manual offers guidance so that you may be effective in this regard. It describes the context in which you will be operating, and it offers direction for your specific duties. It provides an overview of bipolar disorder, the state-of-the-science treatments for bipolar disorder, and our protocol designed to advance the treatment of bipolar disorder. It also describes in some detail the basic competencies you will rely upon in your work with participants. Based on a model called “supportive accountability” you will engage and motivate those enrolled in the study as well as assist them in the utilization of the application.

2

TRAINING SCHEDULE

Training will include reading, instruction, modeling, role play, coaching, and feedback. This training manual and the recommended supplemental articles will provide a basic understanding of the information you will need to know as well as the skills you will need to use. Face-to-face training meetings will offer an opportunity to round out your knowledge base and to develop competencies as a coach.

Preparation

In advance of the full-day workshop, be sure to read the entire training manual including all of the referenced materials. In addition, you will be asked to watch and listen to coaching sessions. This will include at least one training tape of an initial smartphone training session as well as three scheduled calls.

Workshop

The base of training will occur in one full-day workshop. The morning involves review and discussion of the material presented in the training manual. Case examples will be provided to elucidate the material. The afternoon involves learning the coaching skills. All coaches will train to mastery, which is defined as 3 consecutive contacts evaluated at or above 85% satisfactory ratings on the Coaching Rating Scale.

Supervision

Weekly supervision will be provided to coaches. This will include discussion of rating calibrations of initial training sessions and weekly scheduled call using randomly (15% of all contacts) selected audiotapes. The aim is to keep fidelity ratings at or above 85% on the Coach Rating Scale.

3

BASIC FACTS ABOUT BIPOLAR DISORDER

Bipolar disorder is a mood disorder marked by episodes of mania and usually episodes of depression. Individuals may also have episodes with symptoms of both mania and depression called mixed states. You may be familiar with the old term, manic depressive disorder. Individuals with bipolar disorder experience heightened states, normal states (also called euthymic states), and usually depressed states. Bipolar disorder is different from major depressive disorder in that individuals with this depressive disorder do not experience heightened states but rather only one "pole" or depressed states alternating with normal mood states.

Mania

Mania is a state of heightened energy. It is marked by an unusually elevated or irritable mood. It is, however, much more than simply feeling good or 'up'. Mania involves changes in thoughts and behaviors as well.

SYMPTOMS: There are eight core symptoms of mania. Any one symptom does not define mania. Instead, it is exhibiting a constellation of the symptoms all together for an extended period of time that indicates a state of mania. In order to be considered manic, individuals have to exhibit an unusually elevated or irritable mood as well as multiple other symptoms.

- ✓ <u>Unusually elevated or irritable mood</u>
- ✓ Increased activity level
- ✓ Inflated self-esteem/grandiosity
- ✓ Decreased need for sleep
- ✓ More talkative than usual or urge to keep talking
- ✓ Racing thoughts
- ✓ Difficulties with concentration
- ✓ Excessive involvement in pleasurable/risky activities

So what does mania actually look like?

Jason started feeling "righteous". While usually he valued his contribution to work he began thinking he took his job so much more seriously than others, cared more about his work than everyone else, and as a result (in his eyes) performed at a markedly higher level. He was energized and found he required much less sleep. He reported an increase in conflicts with others as he began debating friends online about social and political issues

in a way that was different than normal for him. He usually remained quiet. He was preoccupied with sex and found that others could not keep up with him in conversations. Jackie started finally feeling good about herself again. After a long period of depression, she began interacting with others and working with her church community once again. She had an idea about a workshop they might offer and pitched the idea. It went over quite well. As she began pulling the workshop together, she found herself increasingly excited and energized. Though trying not to get too far ahead of herself, she found herself at odds with the administrative staff, who were having a hard time buying into ideas she had about expanding the workshop into a week-long series. She was sure they simply didn't understand. So she stayed up day and night working on a revised proposal, determined to convince them.

EARLY WARNING SIGNS: The state of mania does not appear without warning. There are identifiable changes that suggest individuals with bipolar disorder are shifting in their mood state. These are called early warning signs. Research identifies many common early warning signs. Note, however, that early warning signs are unique. They vary from individual to individual and may or may not be included in this list. They may be low level symptoms or unique changes in thoughts and behaviors. Common early warning signs include:

✓ Sleep disturbance

✓ More active than usual

✓ More talkative than usual

✓ More social than usual

✓ More irritable/agitated than usual

✓ Increased energy

✓ Increased self-esteem

✓ Racing thoughts

TRIGGERS: Episodes are often, but not always, triggered by events. Common triggers for manic episodes include:

✓ Discontinuing medications
As with other physical and mental disorders, non-adherence is as common as adherence.

✓ Sleep disruptions
This includes schedule shifts as well as changes in the amount of sleep.

✓ Schedule disruptions
Routines that are critical include sleep, seeing others, work, and meal times.

- ✓ Too much involvement in activities
  Individuals with bipolar disorder have a hypersensitivity to rewards and activity involvement.
- ✓ Being stressed
  Threats trigger mania as there is a sensation-seeking response.
- ✓ Critical and intrusive family interactions
  Adverse interpersonal experiences can trigger symptoms.
- ✓ Substance use
  Psychoactive substances can trigger symptoms.

Depression

SYMPTOMS: There are nine core symptoms of depression. Any one symptom does not define depression. Instead, it is exhibiting a constellation of the symptoms all together for an extended period of time that indicates a state of depression. In order to be considered depressed, individuals have to exhibit depressed mood or diminished interest as well as numerous other symptoms.

- ✓ <u>Depressed mood</u>
- ✓ <u>Diminished interested or pleasure in all, or almost all, activities</u>
- ✓ Significant weight loss when not dieting or weight gain
- ✓ Difficulty sleeping or sleeping too much
- ✓ Physical agitation or slow downing
- ✓ Fatigue or loss of energy
- ✓ Feeling worthless or guilty
- ✓ Difficulty concentrating
- ✓ Recurrent thoughts of death or suicide

So what does depression actually look like?

Jason felt low again. He was tearful, afraid to say or do anything. He was still going to work but found that he was preoccupied with what others thought about him. He was certain that somehow he was no longer measuring up. Every day was painful. He would go home at 5:00 p.m. each night relieved to have the stress of the day over. He watched television, ate food purchased at the convenience store, and cried himself to sleep.

Jackie thought she really did not want to live. It seemed as though everything was bad, always was bad, and always would be bad. She considered her life and "realized" that nothing would change. She couldn't hold down a job. Every time she would start out strong but inevitably get depressed and either quit or be fired. She was tired of trying.

EARLY WARNING SIGNS: Like mania, the state of depression does not appear without warning. There are indications that individuals with bipolar disorder are shifting in their mood state. These are called early warning signs. Research identifies many common early warning signs. Note, however, that early warning signs are unique. They vary across individuals and may or may not be included in this list.

- ✓ Sad or anxious mood
- ✓ Less energy than usual
- ✓ Problems concentrating
- ✓ Less interest than usual

- ✓ Negative thinking
- ✓ Withdrawn
- ✓ Sleep disturbance
- ✓ Guilt

TRIGGERS: Episodes are often but not always triggered by events. Common triggers for depressive episodes include:

- ✓ Discontinuing medications
  As with other physical and mental disorders, non-adherence is as common as adherence.

- ✓ Sleep disruptions
  Too little or too much sleep can exacerbate depression.

- ✓ Being stressed
  Loss can trigger a helpless and hopeless response.

- ✓ Critical and intrusive family interactions
  Adverse interpersonal experiences can trigger symptoms.

- ✓ Substance use
  Psychoactive substances can trigger symptoms.

Episodes

It is typical for people to experience changes in mood, thinking, and behavior from day to day. However, when a number of these changes occur at once, are dramatic, and last for several days or more they create distress and dysfunction. This is called a mood episode. There are four types of mood episodes: mania, hypomania, depression, and mixed.

A manic episode is defined as having an elevated, expansive, or irritable mood and <u>three</u> other symptoms. As you can see, mania is a state of being—or constellation of emotional, cognitive, and behavioral experiences. It is more than just "feeling up". These symptoms need to be present for at least one week, unless hospitalized before then, in order to meet the criteria for full-blown mania. Individuals are often out of touch with reality, if not noticeably psychotic (believing things others in their culture and social group usually would not believe).

A hypomanic episode is a less severe mania. While the symptoms are the same there is not significant impairment and it does not last as long. Individuals will be activated but still in touch with reality.

A depressive episode is defined as having a depressed or anhedonic mood and four other symptoms. As you can see, depression is a state of being—or a constellation of emotional, cognitive, and behavioral experiences. It is more than just "feeling down". All of the symptoms need to be present on most days for at least two weeks in order to meet the criteria for full-blown depression. Individuals may or may not be aware that their perceptions are distorted. Even if they are aware of the distortions, they still experience their bleak perceptions as emotionally true (they "feel true").

A mixed episode is marked by symptoms of both mania and depression. Individuals will be activated and dysphoric at the same time.

Diagnoses

The types of mood episodes that occur determine the specific diagnosis. Bipolar I Disorder involves a history of manic episodes and Bipolar II Disorder involves a history of hypomania and a depressive episode. Cyclothymia involves a history of manic and depressive symptoms neither of which ever met the full criteria for mania or depression. Cyclothymia may be less serious, but it can cause much distress and dysfunction.

The *LiveWell* program is targeted toward individuals with Bipolar I Disorder. So the individuals you will be coaching will have had a manic episode or episodes and possibly depressive episodes in the past.

Causes

There is a strong genetic component to bipolar disorder. In fact, bipolar is one of the most heritable mental health problems. The illness involves many biological systems. Laboratory tests reveal changes during mood episodes in sleep physiology, neurotransmitters, neuroendocrine function, and neuropsychological functioning (ability to organize, retain, process, and use information). These changes generally normalize once the mood episode ends.

Genetic risk does not guarantee development of a bipolar disorder. It is not clear why some of those at risk develop the illness while others do not. Stressful life events appear to play a role in the development of the illness in those individuals already at risk. Additionally, an anxious temperament and low social support create risk for depressive episodes.

Course

With bipolar disorder, the first episode of illness is often between 18 and 30 years of age. Over 95% of those with the disorder have multiple mood episodes or recurrences. Untreated manic episodes usually last between 1 to 3 months. Untreated depressive episodes can last 9 to 12 months. Time between mood episodes tends to shorten over time. Repeated medication discontinuation can make the illness harder to treat.

Consequences

Bipolar disorder can cause distress, dysfunction, disability, and disadvantage. It can be particularly disruptive for relationships and work. It is one of the top 10 leading causes of disability worldwide. Individuals with bipolar disorder often have a significantly increased risk for attempting and completing suicide. This is why it is considered a serious mental illness. However, diagnosis is not destiny. Individuals can recover. Individuals with bipolar disorder can live well.

4

TREATMENTS FOR BIPOLAR DISORDER

READ:

Geddes, J. R., & Miklowitz, D. J. (2013). Treatment of bipolar disorder. *Lancet, 381,* 1672-1682.

Pharmacotherapy

Different types of medications help control the symptoms of bipolar disorder. While it is rare that the medications completely eliminate low-level symptoms or recurrences of full-blown episodes, it is clear that medications can reduce symptoms significantly and delay recurrences. There are several types of medication often used to treat bipolar disorder.

MOOD STABILIZERS: Lithium (lithium carbonate) was approved by the FDA in the 1970s to treat the symptom of bipolar disorder. It is a salt that is well proven to reduce the chance of mania and to reduce the risk of suicide.

ANTICONVULSANTS: Anticonvulsants were originally developed to treat seizure disorders. They can also be effective in treating mania and depression. Anticonvulsants used in bipolar disorder include Depakote (valproic acid), Lamictal (Lamotrigine), Tegratol (Carbamazepine), Trileptol (oxcarbazepine), Topamax (topiramate), and Neurontin (gabapentin).

ANTIPSYCHOTICS: Antipsychotic medications also reduce the symptoms of bipolar disorder. Zyprexa (olanzapine), Risperdol (risperadone), Latuda (lurasidone), Seroquel (quetiapine), Abilify (aripiprazole), and Geodone (ziprasidone) are the newer ("atypical") antipsychotic medications that may reduce symptoms of mania and/or depression as well as preventing recurrence of mood episodes.

ANTIDEPRESSANTS: A number of different antidepressant medications are sometimes used to treat individuals with bipolar disorder. It is not recommended that antidepressants be prescribed without a mood stabilizer, anticonvulsant, or antipsychotic for individuals with bipolar disorder as this increases the likelihood of mania and may destabilize mood, sleep, and behaviors. Commonly prescribed antidepressants include Prozac (fluoxetine), Paxil (paroxetine), Zoloft (sertraline), Wellbutrin (bupropion), Effexor (venlafaxine), Lexapro (escitalopram), and Cymbalta (duloxetine).

Psychotherapy

Medications are the first line of treatment for serious mental illnesses, including bipolar disorder. That said, research suggests that when used as an adjunct (or add-on) treatment, psychosocial treatments can reduce the risk for full-blown illness recurrences by an additional fifty percent as well as often decreasing symptoms overall and improving quality of life. This is significant given that individuals with bipolar disorder are at risk for persistent depressive symptoms as well as full-blown episodes of mania, hypomania, depression, and mixed states.

There are three primary, well-developed and tested psychosocial treatments for bipolar disorder. Again, each of these treatments is offered as adjuncts to medications. These therapies reduce recurrences by about 50%, leading to fewer hospitalizations and better functioning. Benefits outstrip what can be attributed to enhanced medication adherence which is one way these therapies improve clinical outcomes in bipolar disorder.

COGNITIVE BEHAVIORAL THERAPY (CBT)

There is evidence that a number of cognitive and behavioral events are related to relapse. The ability to detect and cope with early warning signs is related to recurrences and functioning. Stress often triggers episodes, and recovery is often longer when the episode is precipitated by life events. Events that activate extreme goal striving accelerate mania. Maladaptive beliefs can interact with life events to increase the likelihood of a manic or depressive episode.

The aim is to reduce vulnerability to illness relapses by enhancing adherence to medication recommendations, regulating sleep and activity schedules, identifying and planning for early warning signs, and managing low level mood symptoms (relapse prevention).

FAMILY FOCUSED TREATMENT (FFT)

There is evidence that stressful family environments worsen symptoms and that, conversely, supportive family environments improve outcomes.

Treatment focuses on enhancing communication in the family and problem-solving skills around the illness and home environment. Relapse prevention is also a focus of FFT.

INTERPERSONAL AND SOCIAL RHYTHM THERAPY (IPSRT)

There is evidence that stress and disrupted routines trigger recurrences. Especially troublesome is interpersonal adversity, chaos, sleep cycle changes, and events that promote goal-directed activities. Conversely, regularity in routines and relationship stability is protective in nature.

Treatment focuses on elucidation of links between mood symptoms and quality of social relationships, quality of social roles, and regularity of daily routines. Work is on identifying and stabilizing social rhythms (daily routines, sleep schedule), identifying and managing precipitants of rhythm disruptions, resolving interpersonal problems (e.g., unresolved grief, conflict, transitions, deficits), and mourning losses related to illness (i.e., healthy self).

5

LIVEWELL PROGRAM

A number of research teams are designing behavioral intervention technologies (BITs), such as web and smartphone based applications, to help individuals with bipolar disorder live healthier lives. *LiveWell* is one of these BITS. Key elements include self-management, coordination of care with psychiatrists, and early intervention. Like standard cognitive-behavioral therapies, *LiveWell* provides education about bipolar disorder, medications, lifestyle, coping skills, and using a treatment team. It offers an opportunity for individuals to develop an awareness of early warning signs of illness and to take action in order to avert full blown episodes. Individuals develop a wellness plan that includes a list of available resources, ways they can reduce the risk of becoming ill, and an action plan for all levels of symptoms.

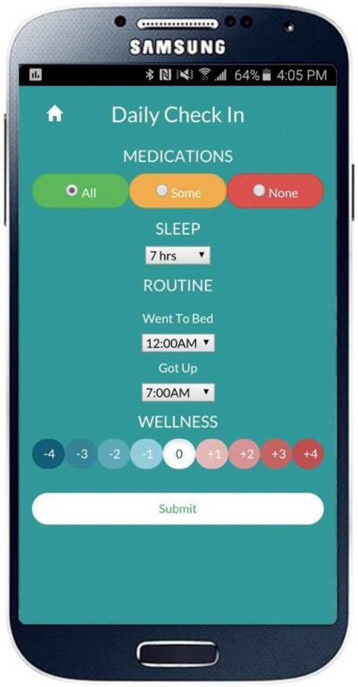

Figure 1

A central feature of the application is a daily check in (Figure 1) and review. Using the phone, participants check-in daily and record their medication use, bedtime and rise time, early warning signs, and wellness rating. In addition, sensors on the phone and a wrist-worn actimeter obtain additional behavioral data including daily patterns of sleep, activity, and social routines. According to their self-report and sensor data, participants receive feedback that directs their attention to areas of concern (medications, sleep, routine, early warning signs, symptoms) and guide them towards solutions. An overview of the components of *LiveWell* is given in the *LiveWell* Design Wireframe (Figure 2).

In sum, the key components of the *LiveWell* application are:

- Foundations: Information necessary to effective self-management is provided.
- Toolbox: Self-assessments and skills for lifestyle, coping with triggers and symptoms, and team building tips.
- Wellness Plan: A customized plan to reduce risk and take action should early warning signs or symptoms arise.
- Daily check-in: A daily entry on medication use, sleep, and wellness rating.
- Daily review: Based on daily check-in, provides information, advice, and encouragement.

Figure 2

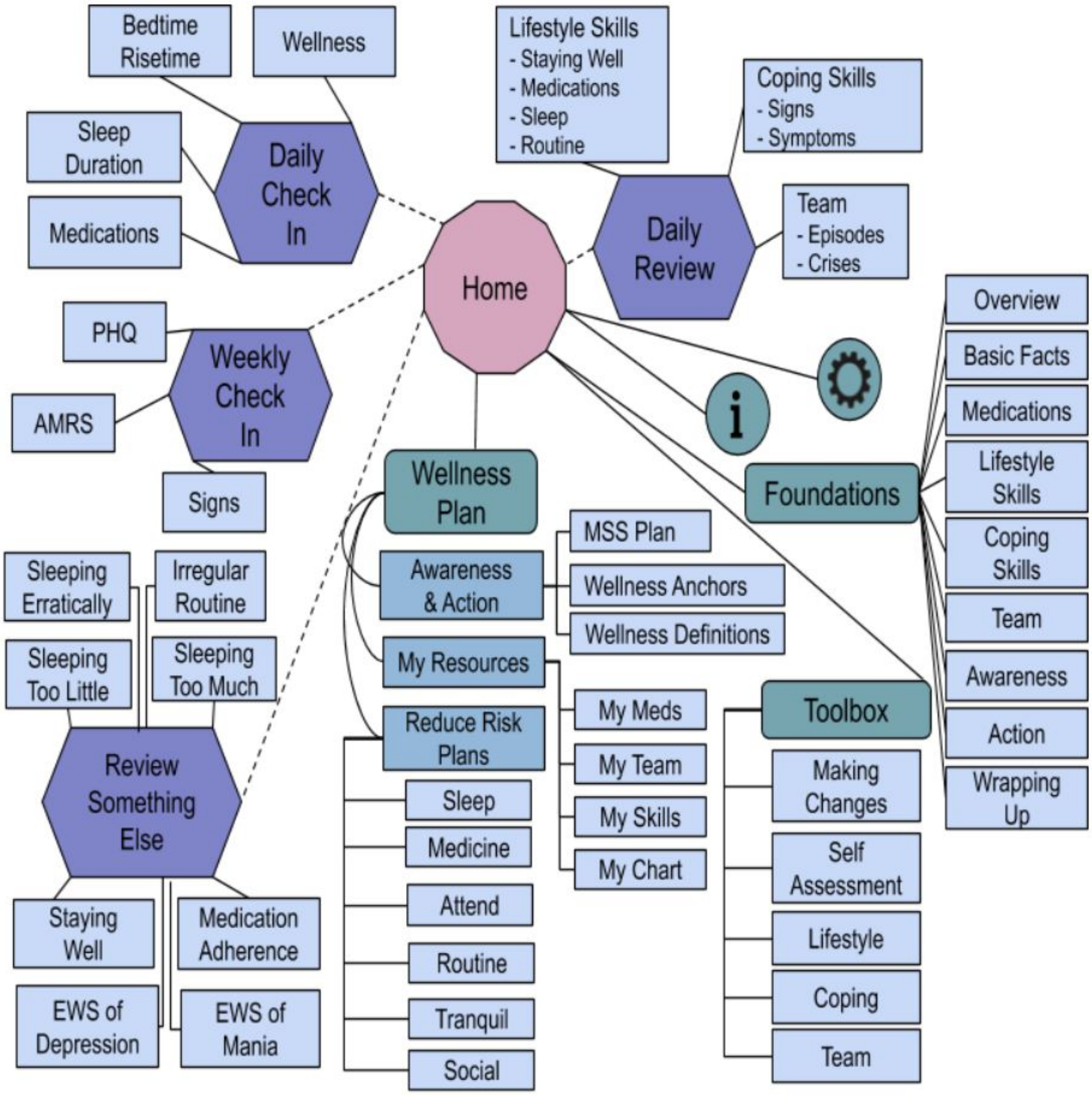

As with standard cognitive-behavioral therapies, the aim is to reduce vulnerability to illness relapses by enhancing adherence to medication recommendations, regulating sleep and activity schedules, identifying and planning for early warning signs, and managing low level mood symptoms, as well as seeking support from clinicians for more serious symptoms. In addition, psychiatrists working with individuals using the application have access to a secure web-based summary of participants' clinical status. The application actively encourages

the participants to contact their psychiatrists when problems arise to facilitate earlier clinical intervention and reduce relapse risk.

Participants receiving the *LiveWell* intervention have one face-to-face meeting with you, the coach, during which you teach them how to use the application and a wrist worn actimeter. At this meeting, you will assist participants in setting goals for sleep duration and routine bed/rise times and anchoring their 9 point wellness rating scale (0 balanced, ±1 normal ups/downs, ±2 new mild symptoms and early warning signs or mild symptoms while recovering, ±3 moderate

Figure 3

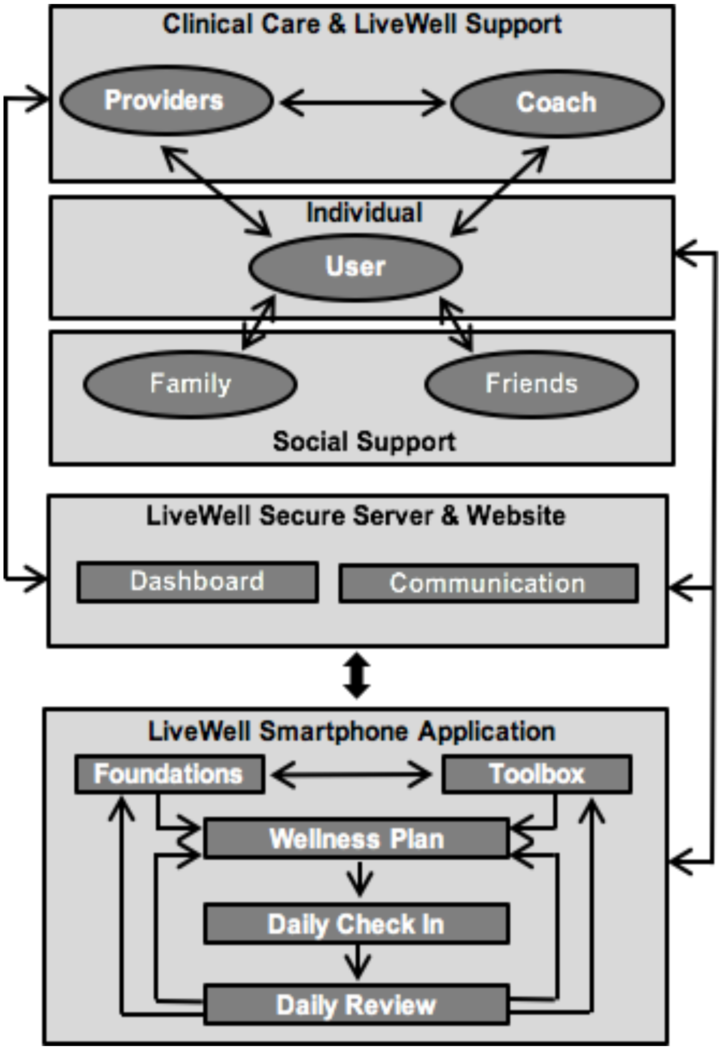

complete 8 standard lessons in 4 weeks and develop a personalized wellness plan. The ongoing core of the application consists of daily check ins and reviews that support utilizing the wellness plan on a regular basis (see participant workflow, Fig. 3). Participants and psychiatrists, if authorized by the participant, also have access to a secure web site for reviewing a clinical status summary. After using the application daily for 4 months, participants read a wrap up lesson to emphasis the termination of requested daily use.

Using 26 main feedback categories, the daily review provides highly tailored feedback to participants based on their current clinical status and

daily rating of wellness, medication adherence, sleep duration, and routine (Figure 4). For instance, if a participant's status is well and their

daily wellness rating is balanced, but their routine is irregular, they will receive supportive feedback on regularizing their routine. Participants are provided with choices about the type of information they would like to view on a given topic providing branch points for multiple feedback conversations which combined with randomization provides diverse feedback. Currently, clinical status is set based on intake evaluation and is updated based on the participants' wellness rating history over the last week. While based on clinical standards for symptom intensity and duration, as well as our survey and face-to-face interviews with providers about when they want to be contacted, we anticipate that the simple rules currently implemented to predict clinical status will require revision during the ongoing development of *LiveWell*.

Figure 4

NC	NP	Feedback Category	Wellness Rating Current Day	Wellness Rating Last 4 Days	Clinical Status	Direction	Action	Goal	RT
1	0	Crisis	Severe Up	NA	Any	Worsening	Hospital	Remain safe	Use Team
1	0	Crisis	Severe Down	NA	Any	Worsening	Hospital	Remain safe	
3	100	Continuing episode	Moderate Up	NA	Unwell	Continuing	Provider/HIT✓	Get help to recover	
3	100	Continuing episode	Moderate Down	NA	Unwell	Continuing	Provider/HIT✓	Get help to recover	
5	25	Improving episode	Mild Up	NA	Unwell	Improving	Provider✓, Continue	Promote recovery	
4	50	Improving episode	Mild Down	NA	Unwell	Improving	Provider✓, Continue	Promote recovery	
5	25	Improving episode	Balanced	NA	Unwell	Improving	Provider✓, Continue	Promote recovery	
10	25	Warning signs	Moderate Up	NA	Not Unwell	Worsening	Sx/Provider/Supports✓, Cope	Prevent deterioration	Use Coping Skills
10	25	Warning signs	Moderate Down	NA	Not Unwell	Worsening	Sx/Provider/Supports✓, Cope	Prevent deterioration	
5	25	Recovering	Mild Up	NA	Recovering	Continuing	Provider✓, Continue	Promote recovery	
5	25	Recovering	Mild Down	NA	Recovering	Continuing	Provider✓, Continue	Promote recovery	
15	375	Prodromal	Mild Up	NA	Prodromal	Continuing	Provider/Supports✓, Cope	Prevent relapse	
15	375	Prodromal	Mild Down	NA	Prodromal	Continuing	Provider/Supports✓, Cope	Prevent relapse	
5	25	Improving recovering	Balanced	NA	Recovering	Improving	Continue	Promote recovery	
5	25	Improving prodrome	Balanced	NA	Prodromal	Improving	Continue	Prevent relapse	
5	25	Warning signs	Mild Up	NA	Well	Worsening	EWS/Provider/Supports✓, Cope	Prevent relapse	
5	125	Warning signs	Mild Down	NA	Well	Worsening	EWS/Provider/Supports✓, Cope	Prevent relapse	
5	25	Taking medications	Balanced	At Risk Severe Meds	Well	High risk	Provider✓, Lifestyle	Lower risk	Use Lifestyle Skills
5	25	Sleeping too little	Balanced	At Risk ↓↓ Sleep	Well	High risk	Provider✓, Lifestyle	Lower risk	
6	25	Sleeping too much	Balanced	At Risk ↑↑ Sleep	Well	High risk	Provider✓, Lifestyle	Lower risk	
60	1500	Taking medications	Balanced	At Risk Meds	Well	Moderate risk	Lifestyle	Lower risk	
20	500	Sleeping too little	Balanced	At Risk ↓ Sleep	Well	Moderate risk	Lifestyle	Lower risk	
15	375	Sleeping too much	Balanced	At Risk ↑ Sleep	Well	Moderate risk	Lifestyle	Lower risk	
25	125	Sleeping erratically	Balanced	At Risk Sleep Erratic	Well	Moderate risk	Lifestyle	Lower risk	
25	125	Irregular routine	Balanced	At Risk Routine	Well	Moderate risk	Lifestyle	Lower risk	
230	8750	Staying well	Balanced	Low Risk	Well	Low risk	Learn more	Build knowledge skills	

FC = Feedback Code; NC = Number of Conversations; NP = Number of Permutations; RT = Resource Type.

Clinical Status State Diagram

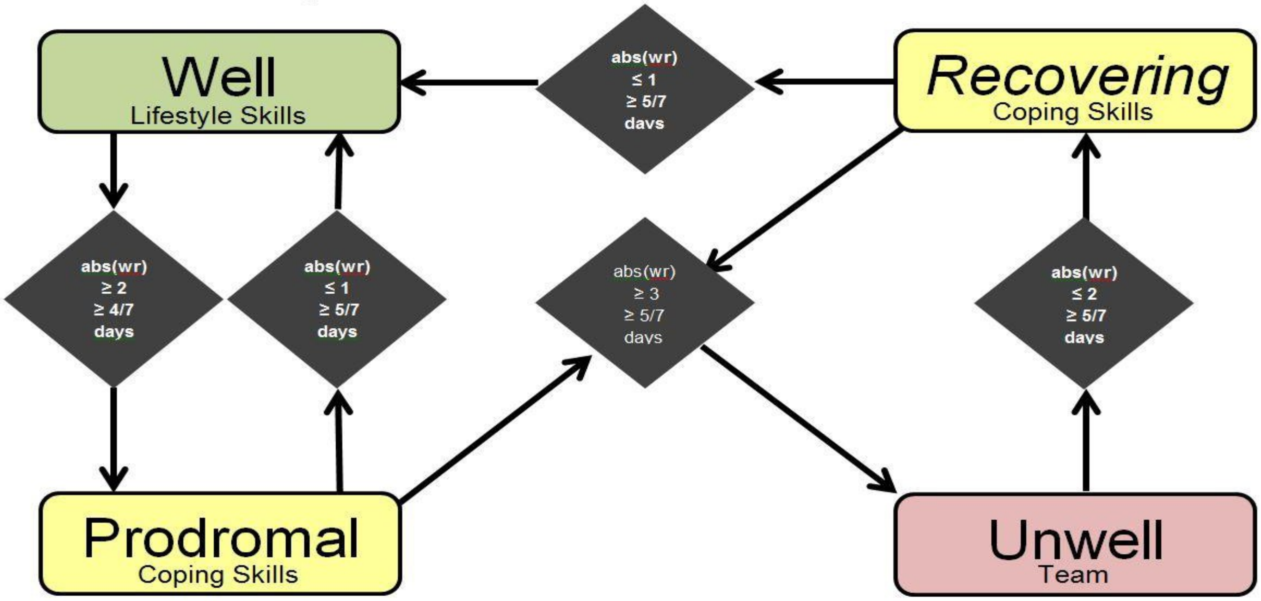

Daily review feedback categories are determined based on participant's current day medication adherence, sleep duration, routine, and wellness rating (wr) and their current clinical status which is updated based on their wellness ratings over the last 7 days as show in the clinical status state diagram.

6

SUPPORTIVE ACCOUNTABILITY

READ:

Mohr, D. C., Cuijpers, P., & Lehman, K. (2011). Supportive accountability: A model for providing human support to enhance adherence to eHealth interventions. *Journal of Medical Internet Research, 13,* e30.

Adherence to BITs is improved with human support. As a result, these interventions are more effective when they incorporate smartphone, email, or other means of contact with a coach. Mohr and his colleagues have developed a model called "supportive accountability" that highlights aspects of support that may be most important to this relationship. The coach is there to help participants maximize their use of the application, or adhere to the program. It is important to note the coach is not a therapist.

Research on adherence (following agreed upon treatment plans with a clinician) to interventions suggests that adherence tends to hover around 50%. Individuals drop out of psychosocial treatments at about this rate, take prescribed medication at about this rate, and follow other medical advice at about this rate. Problems with adherence are not unique to mental health or BITs. The same phenomenon and numbers are found with medical advice as well.

Supportive for BITs means being skilled and collaborative. Being a coach involves facilitating engagement, motivation, and utilization of the *LiveWell* program. The goal is to enhance the participants' investment and adherence to application use.

Accountability for BITs "refers to the implicit or explicit expectation that an individual may be called upon to justify his or her actions or inactions" (Mohr, Cuijpers, & Lehman, 2011). As a coach you will work to increase participants' accountability in regards to specific health behaviors.
Supportive accountability was developed based on theory and research from the fields of organizational psychology, motivation, and computer-mediated communication.

Accountability

Expectations of participants should be clear, made in advance, and offered with a rationale that explains the reasons for the expectations. In a supportive accountability model, expectations should be:

- ➢ Clear
- ➢ Rationale
- ➢ Made in advance
- ➢ Arrived at by consensus

There are two types of expectations. The first is outcome expectations. These are overall goals the coach and participant agree upon. In the *LiveWell* program these outcome goals will likely fall into the categories of reducing symptoms/relapse and improving functioning/quality of life. Process expectations have to do with the effort—or behaviors—that the participant and coach agreed upon that, if made, should in theory bring about the participant's desired outcomes. For example, a participant may aim to reduce symptoms by taking their medications more regularly. Participants have direct control over their behaviors (process) but not results (outcomes). Focusing on process enhances participation while a focus on outcomes tends to have detrimental effects. Process goals should be meaningfully and practically related to outcome goals. The participants' hopes for participating are outcome goals. The goals that participants set each week will be process goals. They focus on behavior.

- ➢ Outcome goals
- ➢ Process goals

It should be made clear that monitoring of behavior is done in the spirit of feedback and learning. Success offers an opportunity to obtain desired outcomes. Lack of success offers an opportunity for self-reflection.

- ➢ Monitoring

Legitimacy

Participants will respond more positively to coaches who are considered legitimate. Coaches need to be seen as experts (knowledgeable) and reciprocal (offering something). They also need to be seen as trustworthy and benevolent.

Bond

Research on therapist bond focuses on liking, trust, and respect. It is thought that having a sense of bond will enhance the effects of accountability.

Motivation

Intrinsic motivation refers to the innate propensity to work towards some goal. This is in contrast to extrinsic motivation, or sources of motivation that are external. Individuals are more apt to engage in behaviors and to sustain behaviors rooted in intrinsic motivation.

- ➢ Be sure application addresses a problem identified by the participant
- ➢ Be sure tasks are engaging and interesting
- ➢ Give choice regarding how tasks are to be completed
- ➢ Offer verbal rewards and acknowledgments
- ➢ Tailor support around participant's level of intrinsic motivation
- ➢ Facilitate "intrinsication" of motivation
- ➢ DON'T: Tangible rewards should be avoided
- ➢ DON'T: Overt and covert pressure is to be avoided

Summary

The adherence and effectiveness of eHealth interventions is improved with human support. Supportive accountability (Figure 5) is a model of human support that integrates theory and data from organizational psychology, motivation theory, and computer-mediated communication.

Accountability of the participant and legitimacy of the coach work together towards an active, personalized, and successful use of *LiveWell*. It is a participant-centered approach designed to help access and implement electronically-based self-management tools.

Figure 5

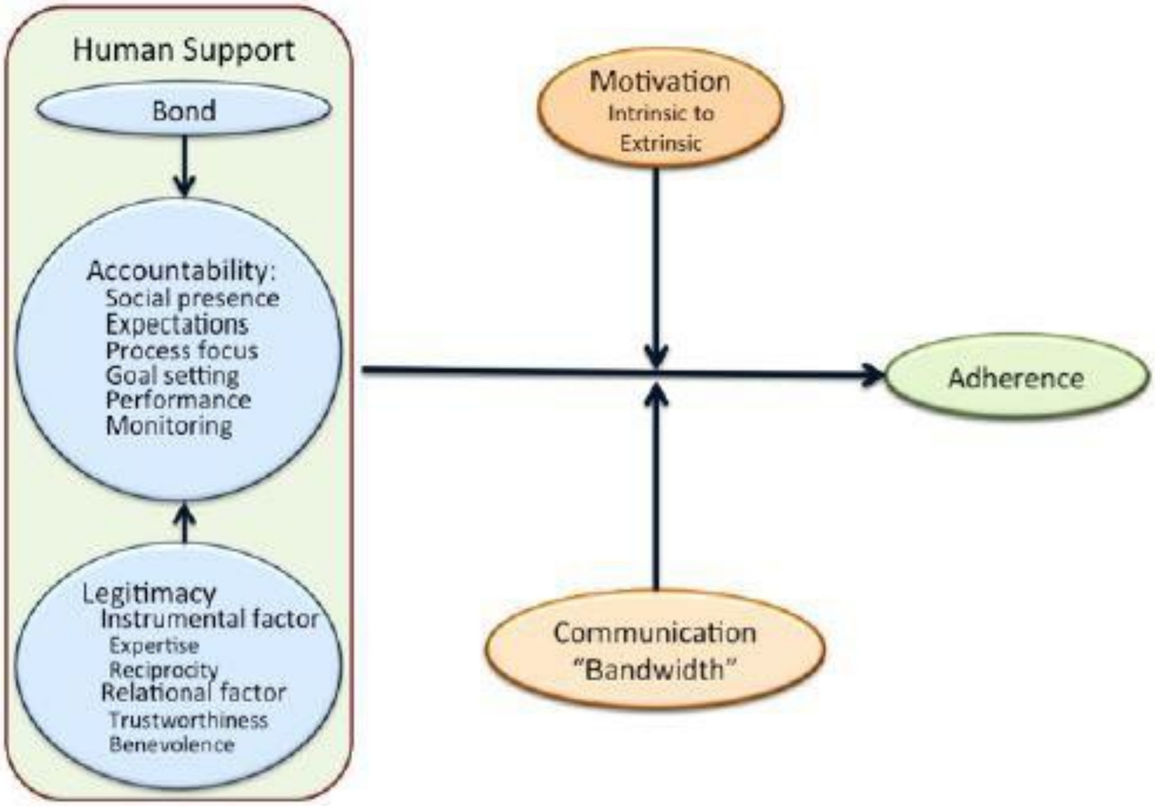

Model of Supportive Accountability (Mohr et al., 2011)

7

BEHAVIOR CHANGE

READ:

Schwarzer, R., Lippke, S., & Luszczynska, A. (2011). Mechanisms of Health Behavior Change in Persons With Chronic Illness or Disability: The Health Action Process Approach (HAPA). *Rehabilitation Psychology, 56,* 161-170.

The implementation of Supportive Accountability relies on strategies of behavior change counseling. Behavior change counseling is designed for non-therapists and is adapted from motivational interviewing. It is a lighter version of this persuasive approach.

> *What does it take to change? Is it just the knowledge that something is good for you? Is that enough? You know from personal experience it is not. Industries are built on helping us eat healthier and exercise more. It is not always easy to do the "right" thing. It in fact is very challenging to commit to and follow through in a sustained manner with health behaviors.*

Research suggests that in order for change to occur, knowledge and motivation together are greater than any resistance (or counter-motivations). Therefore, telling people what to do does not translate to change. In fact, it is more likely to create resistance! There is a term for this. It is called "psychological reactance". That is, when told what to do the impulse is to oppose as autonomy is being challenged. People do not like to feel controlled. This even holds true for the recommendations offered by physicians. Just providing information is not enough to affect change.

In the therapy literature you will find compelling support for the notion of allowing people to establish their own paths, including paths towards health. "People who are committed to goals that they are able to attain and that fit their needs as well as external conditions are more satisfied with their lives, suffer less from psychological symptoms, and show more successful self-regulation…goals and their characteristics are relevant not only as a pathogenic factor that may contribute to the development or maintenance of mental disorders but also as a factor influencing the willingness to be actively involved in therapy" (Michalak & Holtforth, 2006). Furthermore, data suggest that "psychotherapy outcome is enhanced when agreement on therapeutic goals and collaborative involvement (often assessed by participant cooperation, role involvement, and homework compliance) are present during the course of therapy" (see Tyron et al., 2001 for a review). So be a guide with

participants. Don't tell them what to do. Don't collude with bad ideas. Don't confront bad ideas. Be curious. Be supportive. Be an expert. Be collaborative.

In order to be an effective coach, you need to know something about the change process. Readiness to making personal modifications, and in particular changes related to health behaviors, falls along a continuum from not at all, to considering changing, to having changed, and even reverting back to unhealthy ways. Individuals move back and forth through these stages. As a coach you will try to facilitate motivation and volition.

Behavior Change and Maintenance Framework

LiveWell relies on an integration of the available models and data on motivation and change in health behaviors. The framework is non-linear and dynamic, with wellness components, behaviors, and underlying motivational and volitional factors interacting and impacting the entire process of health behavior change.

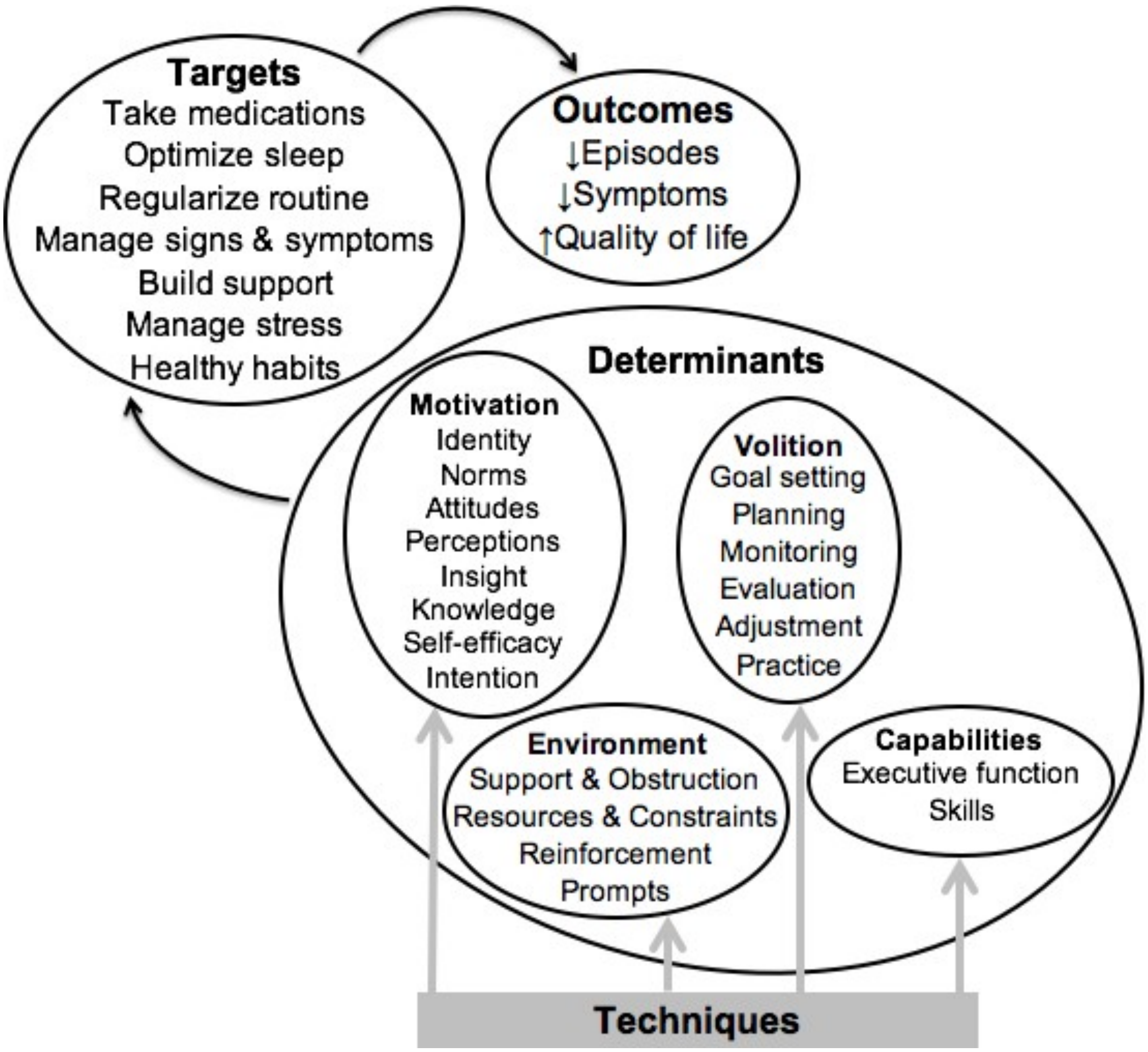

Figure 6

WELLNESS AND BEHAVIOR: In this framework, health and quality of life outcomes are determined by decisions and behaviors involving medications, sleep, routine, and managing early warning signs of illness.

MOTVIATIONAL FACTORS: Knowledge, social norms, perceptions, capabilities, attitudes, supports, and self-efficacy are all known to impact the motivation to initiate, discontinue, and maintain health behaviors. Motivation is the time when participants develop intentions to take action. Initially, they may have no intentions at all to take action. Or they may have motivation and lose it for some reason. All of the following factors contribute to motivation around implementing specific health behaviors (i.e., disease management):

- Knowledge
  Awareness of information necessary to support active participation in management of a health condition.

- Norms
  Beliefs about whether others would approve or disapprove of a behavior (subjective) and desire to comply with others (compliance).
  Beliefs about whether others engage in a behavior (descriptive) and desire to be like others (identification).

- Perceptions
  Beliefs about one's susceptibility to a health condition and the severity of the health condition (risk susceptibility and severity).

- Attitudes
  Beliefs about the tangible costs and benefits (instrumental) and emotional consequences (affective) of a engaging in a behavior.

- Self-Efficacy
  Beliefs about personal ability to perform behaviors needed to achieve an outcome.

- Intention
  Explicit decision to work toward an outcome.

- Identity
  Self-perception of personal characteristics, social roles and types that form a set of standards guiding behavior.

- Insight
  Awareness of having a mental health condition, presence of symptoms and consequences, and need for treatment.

VOLITIONAL FACTORS: The volitional aspect of health behavior change is the time when participants take action. This involves setting concrete achievable goals, planning how to achieve selected goals and how to overcome obstacles and setbacks, as well as exerting control to monitor and evaluate progress, and make adjustments as needed. In particular, action planning involves the when, where, and how of intended action, and coping planning pertains to the anticipation of barriers and ways to overcome them and can be expressed as if-then statements which are called implementation intentions. As with the motivational phase, self-efficacy is a key to action.

- Goal setting
  Identification of a target behavior to work toward to achieve an outcome.

- Planning
  Specific plans for engaging in (task) and for overcoming obstacles (coping) to engaging in a target behavior.

- Monitoring
  Maintaining awareness of engagement in a target behavior.

- Evaluation
  Detecting degree of alignment between actual behavior and target behavior.

- Adjustment
  Based on evaluation, acknowledge success and maintain or refocus current goals and plans or understand problems, identify solutions and make changes in current goals or plans.

- Practice
  Repetition of an action or its elements to learn or improve a capability

Environment Factors:

- Support & Obstruction
  Direct informational, emotional, and/or tangible physical input from others that facilitate or hinder engagement in a behavior.

- Resources & Constraints
  Physical conditions of a situation that support or hinder engagement in a behavior.

- Reinforcement
  Increasing the probability of a behavior by arranging a contingency between the behavior and a consequence that follows the behavior.

- Prompts
  Physical or social stimulus that acts as a reminder to engage in behavior.

Capabilities-based Factors:

- Executive Function
  Cognitive capacities such as response inhibition, mental flexibility, and working memory.

- Skills
  Abilities acquired or developed through practice.

Note that there are two particular internal resources especially important in self-regulation. These are working memory and behavioral inhibition (Heatherton & Wagner, 2010; Muraven & Baumeister, 2000). One must be able to "zoom in" on the task at hand. Interestingly, the ability to control oneself can be literally "exhausted". It is a limited resource. The mind is like a muscle. It gets fatigued with exertion. Exerting self-control, which involves prolonged vigilance and resisting temptation depletes mental reserve. Stress and negative emotions also deplete mental reserve. So self-control efforts, stress, and negative emotions all make it harder to exert self-control. Directing behaviors requires delaying gratification, resisting temptation, perseverance, and problem solving. Studies show that it is possible to improve regulation skills with practice (Fava & Tomba, 2009; Ferguson, Conway, Endersby, & MacLeod, 2009). Keep these things in mind as you coach participants!

Figure 7

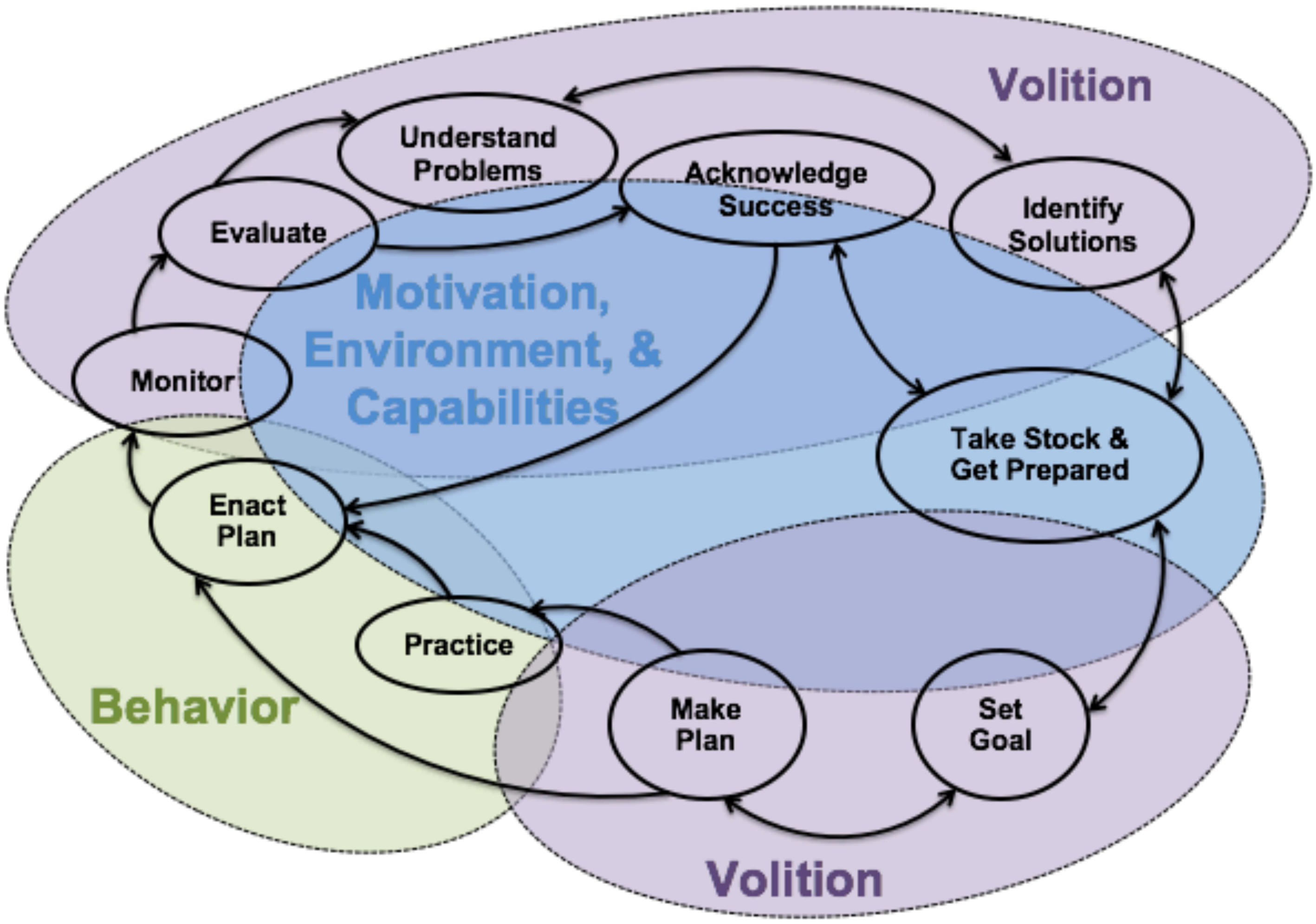

Process of Behavior Change

Research suggests that there are 5 (nonlinear) crucial steps to making lasting behavior changes in life (Figure 7). This is true no matter what the behavior. It could be trying to take medications on a consistent basis, getting a better daily routine, eating better, or exercising more regularly. Specifics about volitional factors are located in the “Making Changes” section of the Toolbox in the *LiveWell* application.

STEP 1: Getting Prepared

Knowledge, capabilities, supports, attitudes, and self-efficacy will impact willingness to initiate changes and stick with the process of making them over time.

- People are more likely to commit to changing when they consider themselves at high-risk for relatively severe consequences of not changing.
- People are more likely to commit to changing when the consequences are important to them, and when the advantages outweigh the disadvantages of making a change.
- People are more likely to proceed with change if they have the internal and external resources to succeed.
- People make changes when they are confident they have what it takes to make it happen.
- People are more likely to commit to change if important people in their life support the changes in question.

STEP 2: Setting Goals

Being clear about goals helps people make changes in life. Being specific and realistic is an important part of this process. For major changes in life, it is best to break down a large goal into manageable steps.

Setting goals is a crucial part of behavior change. Individuals are most motivated when goals are moderate in difficulty. That is, goals should be neither too easy nor too hard. It can be hard to know in advance what is easy and what is hard. Help participants set goals they believe they can achieve with 95% certainty. Early achievement helps with motivation and confidence. When individuals succeed after a few days, weeks, or months they then they can make the goal tougher.

There are two types of goals. Outcomes focus on the results people desire in life. Targets focus on the efforts people make to hopefully produce the desired outcomes. Participants using *LiveWell* will likely have hopes (outcome goals) related to reducing symptoms and episodes resulting from bipolar disorder and improving their quality of life. As a coach, you will be helping them achieve these hopes by supporting them in using the application to work on achieving the behavioral targets (process goals) that are thought to be involved in making interventions like *LiveWell* effective. The targets *LiveWell* focuses on are taking medications, getting the right amount of sleep, keeping a regular routine, managing early warning signs as well as residual symptoms

STEP 3: Developing a Plan

- This involves helping participants identifying what, when, and where they are going to engage in a health behavior. The plan should be action-based, realistic, and involve a time and place. This is called action planning.
- It is important to ensure the participant has the skills, resources, and supports to succeed.
- Anticipate obstacles and ways to overcome them in advance. This is called coping planning.

STEP 4: Monitoring Behavior

Monitoring behavior makes it more likely that participants will effectively make changes. There are two ways to monitor behavior. Self-monitoring, like keep a log, or getting feedback from others helps.

STEP 5. Evaluating Performance

Remember that targets are the <u>efforts</u> (actions) participants planned. How did they do? Did they stick with the plan? Remember that outcomes are the <u>results</u> participants anticipated. Did changes in behavior result in the desired outcomes? Do adjustments need to be made in the targets or outcome expectancies?

8

COACHING SKILLS

The coaching skills in *LiveWell* are based on the models of Supportive Accountability and Behavior Change Counseling. The foundations upon which coaching is built can be translated into four skill domains. Bear in mind these processes, or coaching strategies (stances), when interacting with participants. The keys are participant involvement (collaboration), goal setting and monitoring, "intrinsicizing" motivation, and instrumental and relational legitimacy.

Core Listening Skills

Good listening skills are a part behavioral change counseling and part of being an effective coach. Listen. Demonstrate you're listening. Be sure you understand correctly. Don't assume. Whenever a participant expresses a question or concern, be sure you fully understand the nature of their issue. You can never go wrong by saying "TELL ME MORE…" Remember the acronym OARS to keep in mind the techniques of active listening.

OPEN-ENDED QUESTIONS: These are invitations. Allow room for the participant to craft their narrative. Open-ended questions encourage elaboration. They are non-directive.

Examples:

What was that like?
What were you thinking at the time?
How were you feeling at the time?

AFFIRMATIONS: These are confirmations that you are interested. Show you are listening through verbal and nonverbal encouragements. It urges participants to keep talking.

Examples:

[Lean forward]
[Nod]
Okay
Uh-huh
Hmm

REFLECTIONS: This is a repeating back of the participant's feelings. It shows you are listening and allows you to check that you have accurately understood the participant.

Examples:

You sound frustrated.
You seem happy with how this has gone.
You appear confused.
You sound really down today.
You seem anxious about this.

SUMMARIES: These are recaps. Summarize what the participant has said. Show you are listening and check that you have accurately understood the participant.

Examples:

So you don't understand the check-ins.
You're worried about sharing your plan.
You are committed to trying for a week.
Okay, so you're going to call your doctor.
So you want to go over the daily review.

Domain 1: Agenda Setting and Permission Seeking

Participation in therapeutic endeavors is facilitated by the active involvement of the individuals involved. This includes having input into outcome goals, process goals, and day-to-day topics.

<u>KEY: PARTICIPANT INVOLVMENT</u>

The coach sets an agenda in a way that invites the participant to talk about behavior change.

At the beginning of each encounter, you should explicitly ask the participant's permission to talk about behavior change, making it clear that the participant is not obliged to make any decisions regarding their behavior. That is, you should ask the participant about a willingness to talk about behavior change and give them an opportunity to speak, giving them a choice in the matter!

Example: "Before we start, I'd just like to make it clear that I am not here to tell you what to do or to force you to make decisions you don't feel ready to make. I am here to understand you. We don't have to talk about anything you don't want to talk about. Now I understand you are here to talk about (agenda items). Would it be okay with you if we had a chat about that now?"

The coach demonstrates sensitivity to talking about other issues.

An issue can be anything of concern to the participant, whether it is connected to the behavior in question or not. Be sure to give the participant a choice in what to talk about during each contact. Do not proceed with the consultation discussing what you feel is the most important issue without regard for the participant!

Example: "So, since our last discussion you decided to make some changes in your sleep schedule. That must have been a challenge. I know we're going to focus on that in part, but I'm wondering if there is anything else concerning you that you would like to talk about today as well."

Domain 2: Participant Accountability

Participation in therapeutic endeavors is facilitated by accountability. Accountability involves making one's intentions and results know to another human being. It is facilitated by having a priori clarity, a rationale, and consensus around expectations.

<u>KEY: GOAL SETTING AND MONITORING</u>

The coach facilitates goal setting.

As a coach, be sure to exchange ideas with the participants about how they could change current behavior. Actively encourage the participants to brainstorm a number of strategies that may help them change their behavior. Be sure the participants offer the most ideas; you may make suggestions. Emphasize <u>process</u> (effort) rather than outcomes, but be sure to link process to outcomes.

Example: "So, you are hoping this next week to regularize your sleep schedule. And you said this has been a challenge in the past. What sorts of things might you do in order to make it easier for yourself?"

The coach reviews use of application.

Be sure to elicit information about how the participants think and feel about using the application. Don't assume they have positive or negative feelings. Also be sure not to discount or minimize any problems they are having.

Example: "What are your thoughts and feelings as you look back this week on using the LiveWell application?"

The coach reviews goal achievement.

As a coach, be sure to actively engage the participants in a discussion about the extent to which they met their goals. If participants reached their process goals, explore and link effort to outcome. If the participants did not reach their process goals, focus on lessons learned. Emphasize there is no such thing as failure. Participants either succeed in meeting their process goals or they succeed in learning something.

Example: "So how did things go in terms of implementing a new bedtime routine? ...Okay, so you're saying that it was hard to lie down at 9:00 p.m. every night because you wanted to watch television programs that start at 10:30 p.m. What lessons can you learn from this? How might you take this into account in the week ahead?"

Domain 3: The Why and How of Behavior Change

Motivational strategies facilitate participation in therapeutic endeavors. This involves meeting participants where they are at, highlighting goals, and underscoring behavioral changes that will help them meet their goals. As a coach, follow…do not lead! Motivating participants is not about telling them what to do. *LiveWell* represents a tool that can be useful in helping participants reach their goals. How and when (or if) they use it is ultimately up to them. (Think: you can lead a horse to water, but you can't make it drink.)

KEY: INTRINSICIZE MOTIVATION

The coach elicits how the participant thinks and feels about using the application.

Use a range of (mainly open-ended) questions to draw as much information from the participants as possible about their thoughts and feelings about using the application.

Coach encourages participant to talk about current behavior or status quo.

Encourage the participants to talk freely about what they both like and/or dislike about their current behavior/status quo. You may do this in a variety of ways, for example through using active listening skills, to gain an understanding of the participants' perspective.

Coach encourages participant to talk about behavior change.

Encourage the participants to talk freely about what they feel the positive and negative aspects of behavior change would be for them. You may do this in a variety of ways, for example through using active listening, to gain an understanding of the participants' perspective.

Coach asks questions to elicit how participant thinks and feels about the topic of behavior change.

Use a range of (mainly open-ended) questions to draw as much information from the participants as possible about their thoughts and feelings towards the topic of behavior change.

Coach uses active listening when participant talks about the topic of behavior change.

Uses a range of active listening skills while the participants are talking about behavior change to clarify whether s/he has understood what the participants have said, and/or to encourage the participants to amplify further. Active listening includes open-ended questions, affirmations, reflections, and summary statements (OARS).

Domain 4: The Whole Consultation

Participation in therapeutic endeavors is facilitated by a sense, on the participant's part, that the coach is competent and considerate. This can be demonstrated in many ways.

The first is a sense of trustworthiness and benevolence vis-à-vis displays of optimal levels of warmth, concern, confidence, genuineness, and professionalism. The coach's behavior should convey a sense of integrity, care, and having the participant's best interest at heart. And the second is a sense of expertise and reciprocity. Coaches should be able to provide information about the relevant topics as well as accurately respond to

questions posed by the participant. Be sure to provide sufficient time, attention, and assistance to participant needs.

<u>KEY: INSTRUMENTAL AND RELATIONAL LEGITIMACY</u>

Coach acknowledges challenges about behavior change that the participant faces.

Regularly and explicitly acknowledge the challenges that may be facing the participants. This affirmation is done by focusing on the personal strengths that the participants have in the face of these challenges.

Example: "Goodness, I can see why it is so hard for you to change. You have so much stress to deal with in your life right now. It must be really hard. But even though this is really difficult, you have already begun to make changes to your lifestyle following your recent manic episode, and you've managed to keep them up even though it's tough."

When coach provides information, it is sensitive to participant concerns and understanding.

Try to understand what the participants know and want to know, and also elicits their personal reactions to information provided.

Coach actively conveys respect for participant choice about behavior change.

Openly acknowledges and accept participants' choices even if this does not fit in with your agenda. Do not put any pressure on the participants to change their behavior.

Example: 'At the end of the day, it's your choice. It's up to you if you want to make any changes, and it's about what is realistic and manageable for you. You don't have to do anything you don't want to do.'

Coach and participant *exchange* ideas about the *how* participant could change current behavior.

Actively encourage the participants to brainstorm a number of strategies that may help them change their behavior. With encouragement, the participants should offer the most ideas, and you also make suggestions.

Domain 5: Initial Session Only

During the initial encounter, there are specific things that facilitate overall engagement in the therapeutic endeavor. These include participant involvement in goal setting, clarity of expectations of the participant and coach, and anticipation and planning for obstacles.

- ➢ Obtain information about the participant's reasons for participation.
- ➢ Obtain information about the participant's expectations for the program.
- ➢ Provide participant with information about the application and bipolar disorder.
- ➢ Work with the participant to establish the coach's role.
- ➢ Work with the participant to establish how coach and participant will work together.
- ➢ Obtain information about the participant's challenges towards participation.
- ➢ Elicit enthusiasm and commitment talk from the participa

9

COACHING RESPONSIBILITIES

Remember that the purpose of coaching is to support the participants' use of the *LiveWell* program. This means keeping them engaged and motivated. It also means helping them solve any problems in using the application itself.

You are expected to offer answers about use of the application and to support the participants in using it for their advantage. You are not expected to act as a therapist or clinical advisor. Also, you are not expected to "make" them do anything they are reluctant to do (Figure 8).

See Appendices D through I for the following coaching scripts: Initial Meeting, Scheduled Calls, Ad Hoc Calls, Crisis Protocol, Suicidal Ideation Protocol, and Coach Fidelity Rating Scale.

Initial Meeting

This is a face-to-face session in which you introduce the participant to the telephone and the basic concepts of the *LiveWell* Program. The session focuses on providing information, enhancing motivation, and working towards commitment to specific behaviors vis-à-vis the *LiveWell* application.

Scheduled Calls

There are planned coaching calls at weeks 1, 2, 3, 4, 6, and 16. Generally speaking, these calls are to review material and goal achievement, set new (or continued) goals, and anticipate obstacles to participation and problem solve.

Weeks 1 through 3 focus on reviewing the lessons. Week 4 is longer and is designed to help the participant generate their Wellness Plan. Week 6 is designed simply as a planned check in. The final week (16) is scheduled to review a termination lesson and to wrap-up with the program and coaching relationship.

Ad Hoc Calls

There are a few additional conditions under which coaches will reach out to participants. These include:

- Low application adherence
- Discrepancy in clinical ratings
- Severe symptom ratings (daily and weekly check ins)

The primary goal of these calls is to problem solve. In the case of severe symptom ratings, the goal will be to screen for safety concerns. (Any confirmed concerns are reported to the clinical coordinator or clinician on call, who will conduct further assessments and make clinical recommendations.)

Crisis Protocol

A very specific protocol has been designed to screen participants any time there is concern that they have entered a full-blown manic or depressive episode. Coaches are responsible only for completing the screening tool and reporting the results to the clinical coordinator or clinician on call. Coaches ARE NOT clinically responsible for determining level of risk or providing further clinical care.

Suicidal Ideation Protocol

A very specific protocol has been designed to screen participants any time there is concern that they are in danger of taking their life. Coaches are responsible only for completing the screening tool and reporting the results to the clinical coordinator or clinician on call. Coaches ARE NOT clinically responsible for determining level of risk or providing further clinical care.

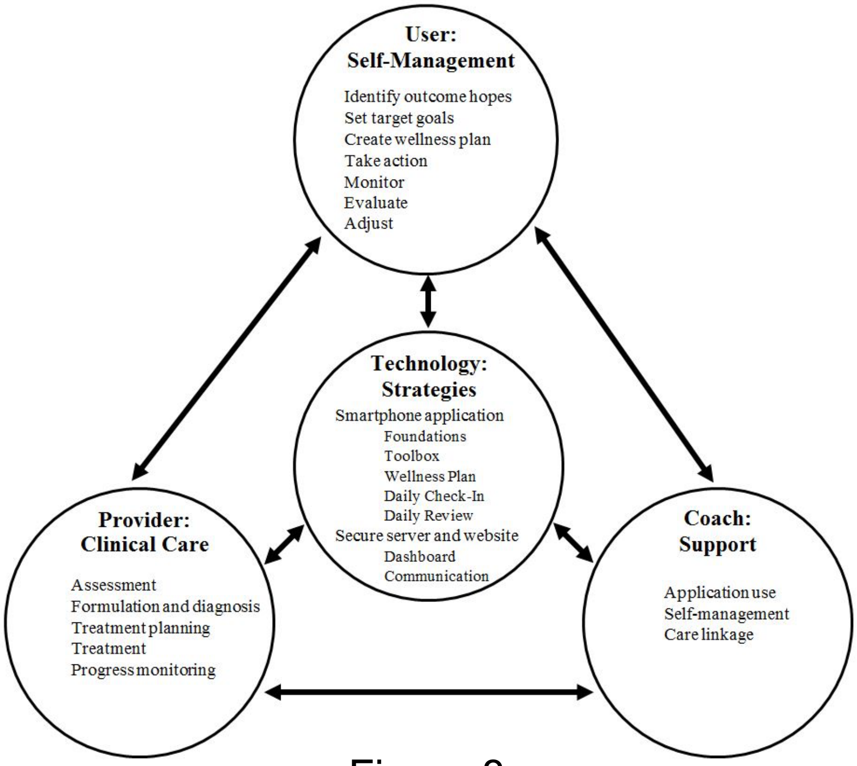

Figure 8

10

WRAPPING UP

A lot of material has been covered in this training manual. Likewise, there is a lot of material in the *LiveWell* application itself. Take your time to familiarize yourself with the basic concepts. You will master it all with time.

Remember, this manual is a starting point for your training. It also serves as a reference guide for you down the road. In addition to the materials included here, you will get face-to-face instruction, watch others, and role play with feedback all prior to starting as a coach. So read, wonder, and ask questions as you go through your training.

We look forward to facilitating your participation as a coach in the *LiveWell* program!

REFERENCES

Geddes, J. R., & Miklowitz, D. J. (2013). Treatment of bipolar disorder. *Lancet, 381,* 1672-1682.

Goulding, E. (2015). LiveWell. [Smartphone application]. Chicago, IL: Northwestern University.

Mohr, D. C., Cuijpers, P., & Lehman, K. (2011). Supportive accountability: A model for providing human support to enhance adherence to eHealth interventions. *Journal of Medical Internet Research, 13,* e30.

Schwarzer, R., Lippke, S., & Luszczynska, A. (2011). Mechanisms of Health Behavior Change in Persons With Chronic Illness or Disability: The Health Action Process Approach (HAPA). *Rehabilitation Psychology, 56,* 161-170.

BASIC FACTS ABOUT BIPOLAR DISORDER

American Psychiatric Association. (2013). Bipolar disorder. In *Diagnostic and Statistical Manual of Mental Disorders V* (pp. 123-154). Washington, DC: American Psychiatric Association Press.

Jones, S. H., Sellwood, W., & McGovern, J. (2005). Psychological therapies for bipolar disorder: The role of model-driven approaches to therapy integration. *Bipolar Disorders, 7,* 22-32.

National Institute of Mental Health. (2014). *Bipolar disorder.* Retrieved from http://www.nimh.nih.gov/health/topics/bipolar-disorder/index.shtml .

TREATMENTS FOR BIPOLAR DISORDER

Frank, E. (2005). *Treating Bipolar Disorder: A Clinician's Guide to Interpersonal and Social Rhythm Therapy.* New York: Guilford Press.

Miklowitz, D. J. (2008). *Bipolar Disorder: A Family-Focused Treatment Approach.* (2nd Ed.) New York: Guilford Press.

National Institute of Mental Health. (2014). *Bipolar disorder.* Retrieved from http://www.nimh.nih.gov/health/topics/bipolar-disorder/index.shtml .

Ramirez-Basco, M., & Rush, A. J. (2005). *Cognitive-Behavioral Therapy for Bipolar Disorders* (2nd ed.) New York: Guilford Press.

SELF MANAGEMENT STRATEGIES

Fava, G. A., & Tomba, E. (2009). Increasing psychological well-being and resilience by psychotherapeutic methods. *Journal of Personality, 77,* 1903-1934.

Ferguson, G., Conway, C., Endersby, L., & MacLeod, A. (2009). Increasing subjective well-being in long-term forensic rehabilitation: Evaluation of well-being therapy. *Journal of Forensic Psychiatry & Psychology, 20,*906–918.

Fletcher, D., & Sarkar, M. (2013). Psychological resilience: A review and critique of definitions, concepts, and theory. *European Psychologist, 18,* 12-23.

Heatherton, T. F., & Wagner, D. D. (2010). Cognitive neuroscience of self-regulation failure. *Trends in Cognitive Sciences, 15,* 132-139.

Hofmann, W., Schmeichel, B. J., &Baddeley, A. D. (2012). Executive functions and self-regulation. *Trends in Cognitive Sciences, 16,*174-180.

Lundahl, B., & Burke, B. L., (2009). The effectiveness and applicability of motivational interviewing: A practice-friendly review of four meta-analyses. *Journal of Clinical Psychology, 65,* 1232-1245.

Martins, R. K., & McNeil, D. W. (2009). Review of motivational interviewing in promoting health behaviors. *Clinical Psychology Review, 29,* 283-293.

Michalak, J., & Holtforth, M. G. (2006). Where do we go from here? The goal perspective in psychotherapy. *Clinical Psychology: Science and Practice, 13,* 346-365.

Rollnick, S., Miller, W. R., & Butler, C. C. *Motivational Interviewing in Health Care: Helping Participants Change Behavior.* New York: Guilford Press.

Tyron, G. S., & Winograd, G. (2001). Goal consensus and collaboration. *Psychotherapy, 38,* 385-389.

Muraven, M., & Baumesiter, R. F. (2000). Self-regulation and depletion of limited resources: Does self-control resemble a muscle? *Psychological Bulletin, 126,* 247-259.

Park, N., Peterson, C., & Seligman, M. P. (2004). Strengths of character and well-being. *Journal of Social and Clinical Psychology, 23,*603-619.

Peterson, C., Ruch, W., Beerman, U., Park, N., & Seligman, M. E. P. (2007). Strengths of character, orientation to happiness, and life satisfaction. *Journal of Positive Psychology, 2,* 149-156.

Quinlan, D., Swain, D. A., & Vella-Brodrick, D. A. (2012). Character strengths interventions: Building on what we know for improved outcomes. *Journal of Happiness Studies, 13,* 1145-1163.

Wilson, K. G., Sandoz, E. K., & Kitchens, J. (2010). The Valued Living Questionnaire: Defining and measuring valued action within a behavioral framework. *The Psychological Record, 60,* 249-272.

II. Application Training Script

LiveWell Application Training Script: Intervention Arm

LWID:____________________ Coach:____________________ Date of session:____________

PREPARE FOR SESSION. Pre-loaded phone/charger, watch/charger, recorder, money, handouts.

1. Introduction

Welcome. Thanks for coming to participate in the *LiveWell* study. I'm [name]. I will be working as a coach to follow along with you while you participate in the study. You have been randomly assigned to the behavioral data collection and self-management arm of the study.

- As we talk today, I'll take some notes
- Audio recording- only study team listens
- Today, about 1 hour
- Go over how to use the equipment
- Go over how to use the self-management application
- May seem like a lot so don't worry about every detail
- Everything we cover today is in Instructions section of app
- You can call or email me with questions as well

Do you have any questions right now?

____

____

____

2. Hopes

Make links between the participant responses and how the application might be useful.

How did you decide to participate in this program?

____

____

____

What would you like to be different at the end of this program?

____

____

____

3. Overview

- *LiveWell* focuses on self-management tools to help decrease mood episodes
- Medications shown reduce risk symptoms and episodes in bipolar disorder
- Most people know about the need for medications in bipolar disorder
- Self-management cuts down symptoms up to 50% compared with medications alone
- Many people do not know about these tools

What do you do to manage bipolar disorder now?

Make links between the participant responses and how the application might be useful.

____

____

____

General Rationale

- Time-limited, 4-month treatment
- Help you learn more about your symptoms
- Identify ways to reduce their likelihood of occurring
- Manage them when they occur
- Hopefully in the future be better prepared to manage bipolar

4. Wellness Rating Scale

- Important part of *LiveWell* is knowing how you're doing each day
- Different people describe this differently
- Before we go to the application, we'll create your personalized wellness rating scale
- Let's review this [HANDOUT 1]
- You will write down 3-5 brief statements for each rating
- Can describe your
    - Moods and emotions
    - Kind of thoughts you have
    - Whether your thinking is fast or slow
    - Your behaviors, sleep, or energy levels
    - Your interests or outlook on life
    - How you relate to others
- I will load anchoring statements on the phone for you so best to keep reminders brief
- Will review in 4 weeks, can update for you if you want to change at anytime

Wellness Scale (-3)

If describing most severe or crisis level of depression, anchor as -4 and return to -3.

On this scale, episodes of depression correspond to feeling moderately down (-3). People usually have:

- Multiple symptoms
- Symptoms that continue day after day
- May have difficulty maintaining their usual activities and routines

Write down a few words that describe your experiences when having a depressive episode.

Ok. Now let's take a look at this list. It that covers the main symptoms of depression [HANDOUT 2]. Are there any symptoms on this list that resonate more with you? If so, adjust your sheet accordingly.

Wellness Scale (-4)

If past hospitalizations due to depression, they might consider what this was like. If they were never suicidal or hospitalized they might consider what would lead to a 911 call or hospitalization.

Let's consider what would be a crisis situation due to depression. This might include:

- Thinking about suicide including making or acting on plans to kill yourself
- Experiencing psychotic symptoms such as delusions or hallucinations
- Being unable to maintain your regular activities, responsibilities, and routines
- Being unable to sleep, eat, or bath regularly
- Engaging in behaviors with serious consequences (risky, dangerous)

Write down a few words or phrases that would describe you at such a time.

Wellness Scale (-2)

On the rating scale, early warning signs of depression correspond to feeling mildly down (-2).

- Many people notice low level symptoms or other signs before a depressive episode
- These changes happen first and often predict an episode of depression
- These kinds of changes are called early warning signs
- Learning to notice and take action helps avoid episodes

People often maintain their usual activities and routines when early warning signs are present.

Write a few words or phrases that describe how you are before an episode of depression. Try to list signs or symptoms that come FIRST for you.

Here is a list [HANDOUT 3] of early warning signs of depression that are fairly common. Are there any on this list that resonate with you that you'd like to add to your sheet?

Wellness Scale (+3)

If describing most severe or crisis level of mania, anchor as +4 and return to +3.

On this scale, episodes of mania correspond to feeling moderately up (+3). People usually have:

- Multiple symptoms
- Symptoms that continue day after day
- May have difficulty maintaining their usual activities and routines

Can you write some words or phrases that describe when you are having a manic episode?

Ok. Now let's take a look at this list that covers the main symptoms of mania [HANDOUT 4]. Are there any symptoms on this list that resonate more with you? If so, adjust your sheet accordingly.

Wellness Scale (+4)

If past hospitalizations due to mania, they might consider what this was like. If they were never hospitalized they might consider what would lead to a 911 call or hospitalization.

Let's consider what would constitute a crisis situation due to a manic episode. This might include:

- Being unable engage in your daily activities, responsibilities, and routines
- Being unable to sleep, eat or bath regularly
- Behaviors with serious consequences (risky, dangerous, fighting, promiscuous, spending)
- Having very poor judgment, making very bad decisions
- Experiencing psychotic symptoms such as delusions or hallucinations

Write down a few words or phrases that would describe your experiences at such a time.

Wellness Scale (+2)

On the rating scale, early warning signs of mania correspond to feeling mildly up (+2).

- Many people notice low level symptoms or other signs before entering a manic episode
- These changes happen first and often predict an episode of mania
- These kinds of changes are called early warning signs
- Learning to notice and take action helps avoid episodes

People often maintain their usual activities and routines when early warning signs are present.

Write a few words or phrases down that describe this how you are before an episode of mania. Try to list signs or symptoms that come FIRST for you.

Here is a list of early warning signs of mania that are fairly common [HANDOUT 5]. Are there any on this list that resonate with you that you'd like to add to your sheet?

Wellness Scale (0)

Let's cover when you are doing well and feeling balanced (0). Write down a few words that describe what it's like for you when you are balanced. Think about:

- Your mood and thoughts
- Your behaviors
- Your personality
- Your interests
- Your outlook on life
- How you relate to others

Wellness Scale (-1)

Next we can talk about times when you feel slightly down (-1). This type of down is:

- A typical response to routine negative event in daily life
- Usually a normal variation in mood, thoughts, or behavior

Write down a few words or phrases that you at these times.

Wellness Scale (+1)

Now think about times when you feel slightly up (+1). This type of up is:

- A typical response to routine positive event in daily life
- Usually a normal variation in mood, thoughts, or behavior

Write down a few words or phrases that you at these times.

5. Home Page

Let's look at the application. Tell me if I'm going too fast or too slow. Also ask questions at any time. But before we begin, let me ask. What has your experience been with android phones? How comfortable are you with technology?

Offer general orientation to operation, navigation, etc. as needed.

Okay, now let's look at the *LiveWell* application. You will see this home page when you open the application. Right now, there are a few buttons:

- Weekly Check In
- Daily Check In
- Foundations
- Toolbox
- Wellness Plan

6. Daily Check Ins

Reminders

Every day application displays a reminder to check in:

- Comes up in tray [SHOW]
- Will be there until you complete *LiveWell* activities for the day

Settings

Let's set when you'd like to receive the first reminder of the day to complete *LiveWell* activities:

- Daily Check In
- Daily Review
- Weekly Check In (on Sundays only)

If not a good time or you don't complete daily check-in:

- Alert icon will stay in tray
- You will get three reminders (set time and 2 and 4 hours after that)
- Can always change notification time (but should be before 8:00 p.m.)

Any questions about how to set when you want to check in or about the reminders?

Let's go over each part of the daily check in. To reduce symptoms and episodes, it is important to take medications regularly, get adequate amounts of sleep, keep a regular schedule or routine, and monitor for early warning signs of depression and mania. We'll set your intentions today for each of these self-care areas.

<u>Medications</u>

- Can be difficult for people to take medications daily
- Record if you took all, some, or none of daily psychiatric medications in the past 24 hours
- Do not need to include PRN medications or medications for other conditions

What is your target for taking your medications for bipolar disorder [aim for 100%]?

____
____
____

<u>Sleep</u>

- American Sleep Foundation recommends: 7 – 9 hours/night, 6 – 10 range normal okay for some
- Enter the number of hours you think you slept each night (not time in bed)
- Don't include naps

What is your target for amount of sleep [aim for 2-hour window between 6 to 10 hours]?

____
____
____

<u>Routine</u>

- Push button to record when you went to bed with the intention of going to sleep (not when you went to sleep but when you tried to get to sleep)
- Push button to record when you got up planning to start your day
  (not when you woke up but when you got out of bed to start your day)
- Be mindful of AM/PM for times selected

What is your target for routine [aim for 1.5-hour window for bedtime and risetime]?

____
____
____

Wellness Scale

- Record wellness rating (for the previous day)
- Don't rate how you are doing just in the moment you are checking in
- Rate how you have been overall the past 24 hours
- Think about the anchors for the scale that you created
- Review your anchors and general definitions from time to time
- Once you press the number, button turns white

Some people with bipolar disorder have what's referred to as mixed episodes. This means depression with some symptoms of mania, or mania with some depressive symptoms at the same time. If you experience this, enter a rating that captures the more prominent symptoms.

What's your target in terms of your wellness rating? [Aim for +1 to -1]
____
____
____

Let's complete the Daily Check In together now.

Any questions?

7. Daily Review

- Comes up automatically after Daily Check In
- Feedback based on how you've been doing
- Recommend you look at every day
- Percentage success for each target past 7 days [SHOW HOVER GOALS]
- Can repeat suggested Daily Review from home screen
- Can Review Something Else

Let's complete the Daily Review together now.

Any questions?

8. Weekly Check Ins

- Comes up Sundays
- Includes standardized questions about depression and mania
- Also early warning signs for depression and mania
- Responses should consider entire past week, not just current day

Let's complete the Weekly Check In together now.

Any questions?

9. Clinical Reporting

LiveWell is equipped to generate weekly clinical reports. It is also equipped to generate notifications whenever you are at risk for having a mood episode or are having a crisis.

You will have access to an online weekly report that displays an overview of how you are doing that looks like this [HANDOUT 6]. You were emailed a link and password. Did you get it? [If not, then take a look.] The reports do not contain your full name or other identifiers, and the website is secure.

You will also get notifications in your Daily Review whenever you are at risk for having a mood episode or are in crisis. Based on your Daily Check In, *LiveWell* may prompt you to call your psychiatrist for problems with taking medications, getting adequate sleep, having early warning signs or symptoms, or having severe symptoms.

You can allow your psychiatrist or other mental health provider access to these two things—your weekly reports and automatic notifications—whenever you are at risk for having a mood episode or are in crisis. Are you comfortable with any of your providers involved in this way?

If yes, complete form for each provider being authorized.

10. Instructions

Now let's briefly look at the instructions page. This section goes over a lot of what we talked about today, since we covered a lot.

11. Refresh

Also on the main page is a refresh button (the two arrows). The application should automatically update your content as we edit things, but if content seems old or is inconsistent with most recent conversation with me, we recommend pressing refresh button before letting me know.

12. Equipment

You might be aware that activity tracking devices are a new trend to help live a healthy lifestyle and maintain a routine. Your study phone and watch will collect behavioral data. We are examining how this information may be useful for helping those with bipolar disorder.

The phone has an application installed, called Purple Robot, that collects data about activity, location, calls, and texts (which does not include information about content or identities).

The pebble watch sends feedback from its sensors to the phone to track sleep and activity level.

- Please wear it 24/7
- Water resistant
- Prefer worn on non-dominant wrist
- Apologize if it feels bulky on wrist
- The pebble band may cause skin irritation. If this occurs, please stop wearing the pebble and contact me. We can provide you with a different type of watchband or you may select a different type of watch band now if you want.

13. Coach's Role

My role is to help you learn to use the application in a way that is most helpful for you. We'll talk each week during the first four weeks and then at week 6 and 16. You can always contact me as well with questions or concerns.

The behavioral and self-report data we are collecting is vital to the success of the study, so I will also be contacting you if check-in, phone, or watch data are not coming in regularly.

What do you think about the coaching calls? How about me checking in with you if the check-in, phone, or watch data are not coming in regularly?

____
____
____

Please note:

- If you have an emergency, you should contact your psychiatrist or call 911.
- Do not discuss your coaching calls or application use with the assessor.

14. Commitment and Goals

Summarize participant's story. Underscore hopes and the ways *LiveWell* can help.

You said you were hoping for [name hopes]. Now that you know more about the application, how do you think the LiveWell program will help you get there?

____
____
____

Beyond concerns talked about [name them if any], what might make it hard to participate in *LiveWell*?

____
____
____

What might you or we do get past these obstacles so you can benefit most from *LiveWell*?

____
____
____

We recommend daily use of the *LiveWell* application. What do you think about that?

____
____
____

<u>18. Wrap Up</u>

In the week ahead we recommend you:

- Read the first two lessons: Overview and Basic Facts
- Complete the Daily Check In and go through the Daily Reviews every day

How does this sound to you?

Do you have any other goals in terms of using the application for the next week?

____
____
____

Ok, so we will talk next week. What is a good time?____

- I appreciate your taking such an active part in learning about the program today
- I think you will find this program useful in helping you stay well.
- Sometimes things come up and people aren't able to use the program as planned
- We can always talk about any problems and I'm happy to help

Do you have any questions for me?

Thank you for coming in today.

Wellness Rating Scale

Wellness	Definition	Anchors
**+4 Severe Up**	Poor judgement. Dangerous behaviors Not sleeping. Hallucinations/delusions.	
**+3 Moderate Up**	Many symptoms day to day. Manic episode probably happening. Difficult to maintain activities/routine.	
**+2 Mild Up**	Some symptoms, early warning signs. Manic episode may be coming. Can still maintain activities/routine.	
**+1 Slight Up**	Response to recent/upcoming good event. Likely normal variation in wellness. Understandable and manageable.	
**0 Balanced**	Neither up nor down. Doing well.	
**-1 Slight Down**	Response to recent/upcoming bad event. Likely normal variation in wellness. Understandable and manageable.	
**-2 Mild Down**	Some symptoms, early warning signs. Depressive episode may be coming. Can still maintain activities/routine.	
**-3 Moderate Down**	Many symptoms day to day. Depressive episode probably happening. Difficult to maintain activities/routine.	
**-4 Severe Down**	Serious ideas about suicide. Immobilized. Dangerous behaviors. Disrupted sleep. Hallucinations/delusions.	

Symptoms of Depression

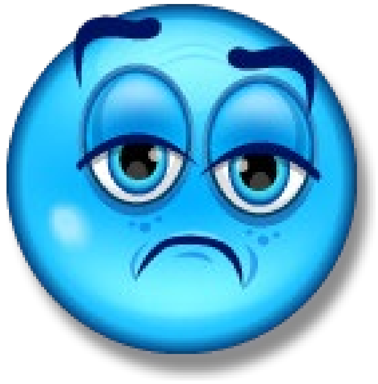

The hallmark of depression is **low mood or diminished interest and pleasure in life**. Other symptoms include:

- ✓ Weight loss or weight gain
- ✓ Sleeping too much or too little
- ✓ Physical agitation or slowing down
- ✓ Fatigue or loss of energy
- ✓ Feeling worthless or guilty
- ✓ Difficulty concentrating
- ✓ Thoughts of death or suicide

Low mood or diminished interest/pleasure plus 4 or more other symptoms on **most days for 2 weeks** is considered a full-blown depressive episode.

Early Warning Signs of Depression

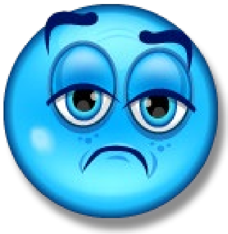

Many people notice low level symptoms or other signs well before entering a depressive episode. These are called early warning signs. Common early warning signs include:

- ✓ Sad or anxious mood
- ✓ Less energy than usual
- ✓ Problems concentrating
- ✓ Less interest than usual
- ✓ Negative thinking
- ✓ Withdrawn
- ✓ Sleep disturbance
- ✓ Guilt

How can you tell if an episode of depression is coming? What changes do you recognize in yourself that lead up to a depressive episode? Are there any signs that you notice **first**?

Symptoms of Mania

The hallmark of mania is a **euphoric or irritable mood**. Other symptoms include:

- ✓ Inflated self-esteem/grandiosity
- ✓ Decreased need for sleep
- ✓ More talkative than usual
- ✓ Racing thoughts
- ✓ Difficulties concentrating
- ✓ Increased activity level
- ✓ Risky activities

Elevated or irritable mood plus 3 or more other symptoms **for a week** is considered a full-blown manic <u>episode</u>.

Early Warning Signs of Mania

Many people notice low level symptoms or other signs well before entering a manic episode. These are called early warning signs. Common early warning signs include:

- ✓ Sleep disturbance
- ✓ More active than usual
- ✓ More talkative than usual
- ✓ More social than usual
- ✓ More irritable/agitated than usual
- ✓ Increased energy
- ✓ Increased self-esteem
- ✓ Racing thoughts

How can you tell if an episode of mania is coming? What changes do you recognize in yourself that lead up to a manic episode? Are there any signs that you notice **first**?

Example of LiveWell Clinical Status Summary

Report Level: Normal

Daily Summary for October 23, 2016

Clinical Status: Well

Recommendation: Keep it up! Stay well. Build knowledge and skills.

	Daily Check In	Goal
**Medication:**	Took All	Take All
**Sleep Duration:**	9 hours	7 - 9 hours
**Bedtime:**	10:30 PM	10:30 PM - 12:00 AM
**Risetime:**	7:30 AM	7:00 AM - 8:30 AM
**Wellness Rating:**	Balanced (0)	-1 to +1

Weekly Summary

Domains	Metric		10/17/16	10/10/16	10/3/16	9/26/16	9/19/16	9/12/16
Weekly Check In Mania	Survey(AMRS) >=6 possible mania		0	0	1	0	3	0
	Early Warning Signs, 8 maximum		0	0	0	0	0	0
Weekly Check In Depression	Survey(PHQ8) >=10 possible depression		2	3	1	1	0	2
	Early Warning Signs, 8 maximum		0	0	0	0	0	0
Daily Check Ins	Days Reported, 7 maximum		5	7	5	7	7	4
Sleep	Duration Min, hours		8.5	7.5	7	7.5	8	8.5
	Duration Mean, hours		9	8.5	8	8	8.5	9
	Duration Max, hours		9	9.5	9	9	9	9
	In Range, %		100	86	100	100	100	50
Routine	In Bedtime Window, %		80	71	80	86	71	50
	In Risetime Window, %		100	100	100	86	86	50
Medications	Adherence, %		100	100	100	93	100	100
Wellness	Moderate Up, %	(+3, Episode)	0	0	0	0	0	0
	Mild Up, %	(+2, Prodromal)	0	0	0	0	0	0
	Well, %	(-1 to +1, Balanced)	100	100	100	100	100	100
	Mild Down, %	(-2, Prodromal)	0	0	0	0	0	0
	Moderate Down, %	(-3, Episode)	0	0	0	0	0	0

LiveWell Program

The LiveWell Program will help you reduce recurrences of depression and mania. While there are a lot of resources available in the application, the key things you will focus on are:

1. Taking medications regularly
2. Getting adequate sleep
3. Developing a good daily routine
4. Managing early warning signs of mood episodes

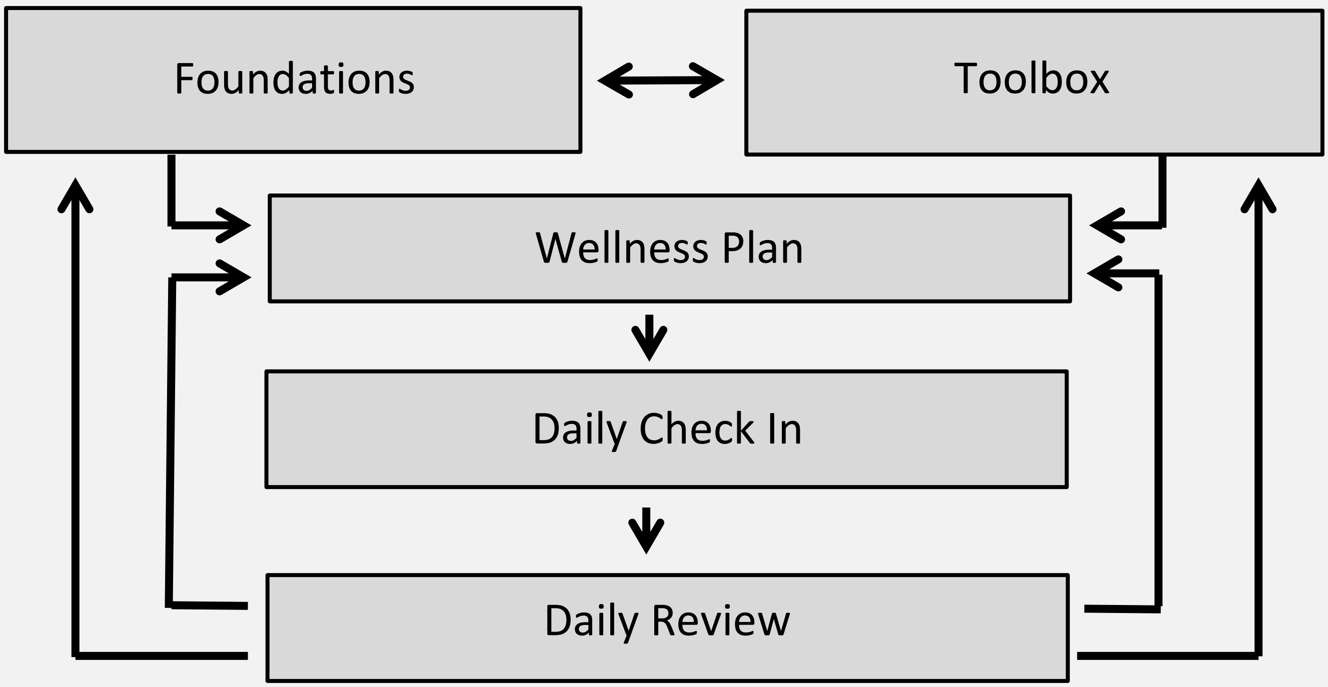

III. Scheduled Call Scripts

LiveWell Week 1 Scheduled Call Script
LWID:____________________ Coach:____________________ Date of session:____________

WEEK 1

PREPARE FOR CALL. Summarize data from the past week.

Dashboard data:

Wellness:	Medications:	Sleep:	Routine:	Check ins:

Coach only:

Feedback category:	Clinical status:	PHQ8:	ASRM:
Weekly goals (last session):			
Hopes (initial session):			

1. Aims

Set clear agenda and invite collaboration.

Hi [participant]. This is your coach, [name], from the *LiveWell* program. I'm calling for our weekly call. It should take about 15 minutes. Is that alright?

Today, we are scheduled to review 2 lessons, how using the daily check in went, and any other goals from last week. Is there anything else you would like to cover?

Any questions or concerns about using the application? Does your personalized content [anchors, medications, psychiatrist] look up to date? Remember that if we ever change things and they don't seem to be updated on your phone, then you can refresh the application to see if that helps. [POINT OUT REFRESH BUTTON]

____

____

____

LiveWell Week 1 Scheduled Call Script
LWID:____________________ Coach:____________________ Date of session:____________

Last week you received the link and password for your weekly reports prior to the phone training. Did you look at them?

____
____
____

2. <u>Review</u>

Remember that success and failure depend on motivation and volition. Keep this in mind in mind while discussing performance.

Let's talk about how things are going. Based on the data I have, it looks like [summarize dashboard]. Does that sound right?

Last time your goal was to [summarize goal]. To what extent were you able to do this?

- <u>If goal met</u>: Good for you! What do you think helped? Do you think that this could have any impact on [summarize hopes]?

- <u>If goal not met</u>: Okay. Let's see what we can learn about this situation. What is your sense of things that got in the way?

____
____
____

3. <u>Goal Setting</u>

Guide participant to set goal based on dashboard data. Prioritize issues in this order: symptoms, medications, sleep, routine, and check ins. If data all within targeted parameters, encourage participant to set goal related to a target or use of the application (e.g., foundations, toolbox, wellness plan). Exchange ideas.

Let's think about your goals for using *LiveWell* in the week ahead. It seems like [summarize highest priority issue] makes the most sense to focus on. Can you think of a goal related to this that you'd like to set for the next week?

Do you think [goal] might help you achieve [hopes]?

____
____
____

Anything that might make it hard to do this? What might help you around this obstacle?

____
____
____

4. **Lessons and Toolbox**

Review lessons. If participant did not look at them, explore what got in the way. Consider whether a motivational or volitional problem. Problem solve together.

Now let's briefly review the lessons. The Overview of the *LiveWell* Program focused on how self-management along with medications can help you stay well; the Basic Facts focused on symptoms, early warning signs, treatment options, and getting support and information about bipolar disorder.

What are your thoughts and feelings about the material? Do you have any questions?

____
____
____

Toolbox: You will become increasingly aware that different parts of the application point you to skills in the Toolbox. Have you had a chance to look at the Toolbox? If you want to navigate there with me now, you'll see there are 5 categories.

- Making Changes: This section provides information on how to get motivated to make personal changes in your life. It also has tips on how to set goals and make plans in a way that increases your chances of success.

- Self-Assessment: This section has a variety of surveys that can help you improve your awareness of symptoms and stress. It also has surveys to help you identify skills, strengths, and supports you might use to get and stay well.

- Lifestyle: This section has tips on how to reduce your risk for symptoms, in other words, ways to boost your resilience.

- Coping: This section describes skills and strategies you can use to manage symptoms of depression and mania.

- Team: This section has tips on how to work effectively with your psychiatrist and personal supports.

How do you think the Toolbox might be helpful?

____
____
____

Material in the Toolbox is there for you to use when you feel like you want or need. Consider taking a closer look. If you find a skill you like, you can choose to add it to your "My Skills" section of the Wellness Plan. Do you want to try adding a skill together now?

5. Close

Sounds like you have a great plan for the week ahead. You are going to try to [goal]. Also read about Medications and Lifestyle Skills before we talk next week.

Can we set a time for next week?____

If anything comes up before then, please feel free to call me. Thanks for talking with me today.

LiveWell Week 2 Scheduled Call Script
LWID:____________________ Coach:____________________ Date of session:____________

WEEK 2

PREPARE FOR CALL. Summarize data from the past week.

Dashboard data:

Wellness:	Medications:	Sleep:	Routine:	Check ins:

Coach only:

Feedback category:	Clinical status:	PHQ8:	ASRM:
Weekly goals (last session):			
Hopes (initial session):			

1. **Aims**

Set clear agenda and invite collaboration.

Hi [participant]. This is your coach, [name], from the *LiveWell* program. I'm calling for our weekly call. It should take about 15 minutes. Is that alright?

Today, we are scheduled to review 2 lessons, how using the daily check in went, and any other goals from last week. Is there anything else you would like to cover?

Any questions or concerns about using the application? Does your personalized content [anchors, medications, psychiatrist] look up to date?

____

____

____

2. **Review**

Remember that success and failure depend on motivation and volition. Keep this in mind in mind while discussing performance.

LiveWell Week 2 Scheduled Call Script		
LWID:____________________	Coach:____________________	Date of session:____________

Let's talk about how things are going. Based on the data I have, it looks like [summarize dashboard]. Does that sound right?

Last time your goal was to [summarize goal]. To what extent were you able to do this?

- If goal met: Good for you! What do you think helped? Do you think that this could have any impact on [summarize hopes]?

- If goal not met: Okay. Let's see what we can learn about this situation. What is your sense of things that got in the way?

____
____
____

3. Goal Setting

Guide participant to set goal based on dashboard data. Prioritize issues in this order: symptoms, medications, sleep, routine, and check ins. If data all within targeted parameters, encourage participant to set goal somehow related to a target or use of the application (e.g., foundations, toolbox, wellness plan). Exchange ideas.

Let's think about your goals for using *LiveWell* in the week ahead. It seems like [summarize highest priority issue] makes the most sense to focus on. Can you think of a goal related to this that you'd like to set for the next week?

Do you think [goal] might help you achieve [hopes]?

____
____
____

Anything that might make it hard to do this? What might help you around this obstacle?

____
____
____

4. Lessons and Wellness Plan

Review lessons. If participant did not look at them, explore what got in the way. Consider whether a motivational or volitional problem. Problem solve together.

**LiveWell Week 2 Scheduled Call Script**
LWID:____________________ Coach:____________________ Date of session:____________

Now let's briefly review the lessons. The <u>Medications</u> provided basic information about medications and how to overcome common barriers to using medications effectively; and the <u>Lifestyle Skills</u> focused on how to promote wellness through sleep, medications, attending, routine, tranquility, and socialization.

What are your thoughts and feelings about the material? Do you have any questions?

____

____

____

<u>Wellness Plan</u>: After the phone training I updated your personalized anchors for the wellness rating scale that you are using in the Daily Check In. These anchors are in the Wellness Plan. Let's navigate there now and make sure everything looks okay. You'll notice the Wellness Plan has 3 sections.

- <u>My Resources</u>: This section lists your medications, team, charts, and skills. You can use charts to help you monitor your goals. My Skills keeps anything you have saved from the Toolbox.

- <u>Reduce Risk</u>: This section has a table for SMART. You can click on a button to learn about related skills. Your personal goals are already in there for medications, sleep, and routine. We will personalize other sections of SMARTs in a couple of weeks. So think about what might be important for you in terms of your lifestyle.

- <u>Awareness & Action</u>: Here we can see the standard definitions, your anchors (which can be updated), and a standard relapse prevention plan. We will personalize your plan in a couple of weeks.

You will learn about developing your Wellness Plan at the end of the Foundations. What do you think about the Wellness Plan? Any questions?

____

____

____

5. <u>Close</u>

Sounds like you have a great plan for the week ahead. You are going to try to [goal]. Also read about Coping Skills and Using a Team before we talk next week.

Can we set a time for next week?____

If anything comes up before then, please feel free to call me. Thanks for talking with me today.

LiveWell Week 3 Scheduled Call Script
LWID:____________________ Coach:____________________ Date of session:____________

WEEK 3

PREPARE FOR CALL. Summarize data from the past week.

Dashboard data:

Wellness:	Medications:	Sleep:	Routine:	Check ins:

Coach only:

Feedback category:	Clinical status:	PHQ8:	ASRM:
Weekly goals (last session):			
Hopes (initial session):			

1. Aims

Set clear agenda and invite collaboration. Check status of provider participant. (a) If participant consented and provider enrolled, let participant know. (b) If participant consented but provider not yet enrolled, let participant know we will not be continuing to reach out but they are free to do so. (c) If participant did not consent, revisit their interest in having their provider participate.

Hi [participant]. This is your coach, [name], from the *LiveWell* program. I'm calling for our weekly call. It should take about 15 minutes. Is that alright?

Today, we are scheduled to review 2 lessons, how using the daily check in went, and any other goals from last week. Is there anything else you would like to cover?

Any questions or concerns about using the application? Does your personalized content [anchors, medications, psychiatrist] look up to date?

____
____
____

**LiveWell Week 3 Scheduled Call Script**		
LWID:____________________	Coach:____________________	Date of session:____________

Before we get into things, let's touch base about your provider. [Summarize status of provider participation.] What do you think about this?

____

____

____

2. **Review**

Remember that success and failure depend on motivation and volition. Keep this in mind in mind while discussing performance.

Let's talk about how things are going. Based on the data I have, it looks like [summarize dashboard]. Does that sound right?

Last time your goal was to [summarize goal]. To what extent were you able to do this?

- If goal met: Good for you! What do you think helped? Do you think that this could have any impact on [summarize hopes]?

- If goal not met: Okay. Let's see what we can learn about this situation. What is your sense of things that got in the way?

____

____

____

3. **Goal Setting**

Guide participant to set goal based on dashboard data. Prioritize issues in this order: symptoms, medications, sleep, routine, and check ins. If data all within good targeted parameters, encourage participant to set goal somehow related to a target or use of the application (e.g., foundations, toolbox, wellness plan). Exchange ideas.

Let's think about your goals for using *LiveWell* in the week ahead. It seems like [summarize highest priority issue] makes the most sense to focus on. Can you think of a goal related to this that you'd like to set for the next week?

Do you think [goal] might help you achieve [hopes]?

____

____

____

Anything that might make it hard to do this? What might help you around this obstacle?

____
____
____

4. Lessons

Review lessons. If participant did not look at them, explore what got in the way. Consider whether a motivational or volitional problem. Problem solve together.

Now let's briefly review the lessons. The Coping Skills provided basic information on how to dial up when depressed and dial down when manic; and the Using a Team Effectively focused on building supports, good rapport with your psychiatrist, and identifying a hospital should the need arise.

What are your thoughts and feelings about the material? Do you have any questions?

____
____
____

5. Close

Sounds like you have a great plan for the week ahead. You are going to try to [goal]. Also read about Awareness and Action before we talk next week.

Can we set a time for next week?____

If anything comes up before then, please feel free to call me. Thanks for talking with me today.

LiveWell Week 4 Scheduled Call Script
LWID:____________________ Coach:____________________ Date of session:____________

WEEK 4

PREPARE FOR CALL. Summarize data from the past week.

Dashboard data:

Wellness:	Medications:	Sleep:	Routine:	Check ins:

Coach only:

Feedback category:	Clinical status:	PHQ8:	ASRM:
Weekly goals (last session):			
Hopes (initial session):			

1. Aims

Set clear agenda and invite collaboration.

Hi [participant]. This is your coach, [name], from the *LiveWell* program. I'm calling for our weekly call. It should take about 30 minutes. Is that alright?

Today, we are scheduled to review 2 lessons, how using the daily check in went, and any other goals from last week. We will also complete your personal Wellness Plan. Is there anything else you would like to cover?

Any questions or concerns about using the application? Does your personalized content [anchors, medications, psychiatrist] look up to date?

____

____

____

2. Review

Remember that success and failure depend on motivation and volition. Keep this in mind in mind while discussing performance.

Let’s talk about how things are going. Based on the data I have, it looks like [summarize dashboard]. Does that sound right?

Last time your goal was to [summarize goal]. To what extent were you able to do this?

- If goal met: Good for you! What do you think helped? Do you think that this could have any impact on [summarize hopes]?

- If goal not met: Okay. Let’s see what we can learn about this situation. What is your sense of things that got in the way?

____
____
____

3. **Lessons**

Review lessons. If participant did not look at them, explore what got in the way. Consider whether a motivational or volitional problem. Problem solve together.

Now let’s briefly review the lessons. The Awareness section focused on learning about your personal early warning signs and symptoms of depression and mania. The Action section offered guidance about how to manage mild, moderate, and severe symptoms.
What are your thoughts and feelings about the material? Do you have any questions?

____
____
____

4. **Wellness Plan**

Okay. Let’s talk about your personal Wellness Plan. I’ll enter what we come up with into the application within the next 24 hours. Go ahead and get your study phone out or put me on speaker if you’re talking on the study phone. Go to the Wellness Plan section.

My Resources

First, let’s take a look at My Resources. You’ll see four buttons.

- Medications: Look at Medications. Are they correct? We can change these any time.

**LiveWell Week 4 Scheduled Call Script**
LWID:_____________________ Coach:____________________ Date of session:____________

Medication	Dosage	Frequency

- Team: Now take a look at Team. Right now it should have your psychiatrist's name and phone number. Is this correct? Who else will be a support? Anyone you'd like to add?

Role	Name	Number
Psychiatrist		
Therapist		
Pharmacy		
Hospital		
Family		
Friends		
Other		

- Skills: My Skills will populate automatically for you whenever you read about a skill in the Toolbox and select to save it into your Wellness Plan.

- Charts: My Charts will also automatically populate for you based on what you report in your daily check-ins.

Reduce Risk

Review and create goals for each area. Anticipate most likely barriers and then ways to get back on track (i.e., if/then statements).

First let's look at the most important areas, which we've already been working on: Sleep, Medications, and Routine. You'll see your targets in there that we set during the phone training. Do these still seem reasonable?

What is the most likely way you might get off track with [each target separately]? What will you do to get back on track with [each target separately]?

Now let's look at Attend, Tranquil, and Social. Let's come up with some goals and some ideas to stay on track. What helps you stay well in terms of [each target separately]?

What is the most likely way you might get off track with [each target separately]? What will you do to get back on track with [each target separately]?

LiveWell Week 4 Scheduled Call Script
LWID:____________________ Coach:____________________ Date of session:____________

Area	Goal
Sleep	
Medications	
Attend	
Routine	
Tranquil	
Social	

When might you refer to this section of your application? When might it be most useful?

___

___

___

Awareness and Action

Now let's take a look at awareness and action. First take a look at your anchors. You came up with those during the application training when you came in and met with me.

Based on what you have learned about bipolar disorder, and about yourself, you may want to make changes. Is there anything you'd like to add, remove, or re-word?

LiveWell Week 4 Scheduled Call Script

LWID:____________________ Coach:____________________ Date of session:____________

Wellness	Anchors
+4 Severe Up	
+3 Moderate Up	
+2 Mild Up	
+1 Slight Up	
0 Well	
-1 Slight Down	
-2 Mild Down	
-3 Moderate Down	
-4 Severe Down	

LiveWell Week 4 Scheduled Call Script

LWID:____________________ Coach:____________________ Date of session:____________

Now take a look at Plan. There are some generic suggestions in there right now. Let's change these to suit you. Pick one, two, or three things you might do when…

Wellness	Action
+4 Severe Up	
+3 Moderate Up	
+2 Mild Up	
+1 Slight Up	
0 Well	
-1 Slight Down	
-2 Mild Down	
-3 Moderate Down	
-4 Severe Down	

When might you refer to this section of your application? When might it be most useful?

____

____

____

5. <u>Close</u>

I will update your application with the information we discussed today. Please let me know if anything looks incorrect.

So from this point forward, we recommend you use your Wellness Plan on a regular basis. The Daily Check Ins and Daily Reviews will help you do this. But be sure you're on track knowing how you want to Reduce Risk when you are doing well and Take Action whenever you have symptoms.

Based on your data today [highlight priority from dashboard], how might you use the application in the week ahead?

Okay, so we will talk in two weeks. Can we set a time now? ________________________

If anything comes up before then, please feel free to call me. Thanks for talking with me today.

LiveWell Week 6 Scheduled Call Script
LWID:____________________ Coach:____________________ Date of session:____________

WEEK 6

PREPARE FOR CALL. Summarize data from the past week.

Dashboard data:

Wellness:	Medications:	Sleep:	Routine:	Check ins:

Coach only:

Feedback category:	Clinical status:	PHQ8:	ASRM:
Weekly goals (last session):			
Hopes (initial session):			

1. **<u>Aims</u>**

Set clear agenda and invite collaboration.

Hi [participant]. This is your coach, [name], from the *LiveWell* program. I'm calling for our weekly call. It should take about 15 minutes. Is that alright?

Today, we are scheduled to review how daily check ins and using your wellness plan went for you. Is there anything else you would like to cover?

Any questions or concerns about using the application?

____
____
____

2. **<u>Review</u>**

Remember that success and failure depend on motivation and volition. Keep this in mind in mind while discussing performance.

LiveWell Week 6 Scheduled Call Script
LWID:____________________ Coach:___________________ Date of session:___________

Let's talk about how things are going. Based on the data I have, it looks like [summarize dashboard]. Does that sound right?

Last time your goal was to check in daily and use your wellness plan. To what extent were you able to do this?

- If goal met: Good for you! What do you think helped? Do you think that this could have any impact on [summarize hopes]?

- If goal not met: Okay. Let's see what we can learn about this situation. What is your sense of things that got in the way?

____
____
____

3. **Goal Setting**

Guide participant to set goal based on dashboard data. Prioritize issues in this order: symptoms, medications, sleep, routine, and check ins. If data all within targeted parameters, encourage participant to set goal somehow related to a target or use of the application (e.g., foundations, toolbox, wellness plan). Exchange ideas.

Now that you've had some time with it, what do you think about your Wellness Plan?

Is there anything you want to change?

____
____
____

4. **Close**

Okay. So from this point forward, we recommend you continue using your Wellness Plan. The Daily Check-Ins and Daily Reviews will help you do this. Be sure you're on track knowing how you want to Reduce Risk and Take Action whenever you have symptoms.

How do you think this might help you?

Is there anything that might make it hard to do this? What might help around this obstacle?

____
____
____

We won't talk again until your last week of actively using the application daily, which is about 2.5 months from now. That last week, a 9th foundation lesson called "Wrapping Up" will be highlighted in white to remind you to read it.

Also remember that if you would like to review a weekly summary of your daily check in data, you can do that using the website along with your username and password.

I will call you to schedule a time to talk as we get closer. While we won't have regularly scheduled calls after today, remember that you can always get help from me with using the application by calling or emailing. Also, if you would like to change any of your personalized content, please contact me.

Remember that I will be reaching out if it looks like there are problems with the watch or phone, or if it appears you are not using the application regularly.

Thanks for talking with me today.

LiveWell Week 16 Scheduled Call Script
LWID:____________________ Coach:____________________ Date of session:____________

WEEK 16

PREPARE FOR CALL. Summarize data from the past week.

Dashboard data:

Wellness:	Medications:	Sleep:	Routine:	Check ins:

Coach only:

Feedback category:	Clinical status:	PHQ8:	ASRM:
Weekly goals (last session):			
Hopes (initial session):			

1. **<u>Aims</u>**

Set clear agenda and invite collaboration.

Hi [participant]. This is your coach, [name], from the *LiveWell* program. I'm calling for our final check in. First I'd like to congratulate you on sticking with the application for four months. How does it feel?

Today, we are scheduled to review the wrap up lesson, how using the application went, and to what extent the application helped you meet your hopes. It should take about 15 minutes.

Is there anything else you would like to cover?

____

____

____

2. **<u>Review</u>**

Remember that success and failure depend on motivation and volition. Keep this in mind in mind while discussing performance.

Let's talk about how things are going. Based on the data I have, it looks like [summarize dashboard]. Does that sound right?

Last time your goal was to [summarize goal]. To what extent were you able to do this?

- If goal met: Good for you! What do you think helped? Do you think that this could have any impact on [summarize hopes]?

- If goal not met: Okay. Let's see what we can learn about this situation. What is your sense of things that got in the way?

____
____
____

3. Lesson

The Wrapping Up lesson offered a summary of the program, asked some questions about how the program worked for you, and provided some questions about how you might best manage your bipolar disorder in the future.

4. LiveWell Program

Did the program help you reach your hopes of [summarize hopes]?

____
____
____

Was anything especially helpful?

____
____
____

What do you plan to do in the future to maintain gains and make further gains?

____
____
____

What do you plan to do in the future should you have any setbacks?

____
____
____

5. **Close**

I appreciate your taking such an active part in using the application. It has been really nice working with you. Do you have any final concerns to discuss?

____
____
____

After this point, use of the application is optional. Would you like to continue to receive notifications to check in daily? [If no, remind the participant that they can contact you at any time in the future to turn notifications back on.]

Please carry the phone and wear the watch daily. I'll call you if there seems to be a problem with the watch or phone. We will be in touch to schedule an appointment to return the equipment.

Thanks for talking with me today. I wish you all the best moving forwards.

IV. Scheduled Call Tips

SYMPTOMS

1. EWS

Read: Foundations → Basic Facts → Early Warning Signs (Depression or Mania as relevant)
Implement: Wellness Plan → Awareness and Action (-2 or +2 as relevant)
Practice: Toolbox → Coping → Dial (Up or Down as relevant)
ALSO: Be sure to complete EWS checklist (Toolbox → Self-assessment), call providers, call supports

2. Subsyndromal

Read: Foundations →Basic Facts → Symptoms (Depression or Mania as relevant)
Implement: Wellness Plan → Awareness and Action (-2/3 or +2/3 as relevant)
Practice: Toolbox → Coping → Dial (Up or Down as relevant)
ALSO: Be sure to complete symptom checklist (Toolbox → Self-assessment), call providers, call supports

3. Unwell

Read: Foundations → Basic Facts → Symptoms (Depression or Mania as relevant)
Implement: Wellness Plan → Awareness and Action (-3 or +3 as relevant)
Practice: Toolbox → Coping → Team
ALSO: Be sure to call provider, consider a higher level/intensity of treatment

MONITORED AND OTHER TARGETS

4. Motivational

Read: Foundations → Lifestyle (Medications, Sleep, or Routine as relevant)
Review: Wellness Plan → Lifestyle (Medications, Sleep, or Routine as relevant)
Complete: Toolbox → Making Changes → Get Prepared

5. Volitional

Read: Foundations → Lifestyle (Medications, Sleep, or Routine as relevant)
Review: Wellness Plan → Lifestyle (Medications, Sleep, or Routine as relevant)
Complete: Toolbox → Making Changes → Evaluate Performance

6. Revise target

As a rule of thumb, revise goals that have not been met for two weeks in a row. Work with patient to aim for a 90% chance of success. Refer together to Toolbox → Making Changes → Set Goal and <u>revise target with participant during the call</u>.

Read: Foundations → Lifestyle (Medications, Sleep, or Routine as relevant)
Review: Wellness Plan → Lifestyle (Medications, Sleep, or Routine as relevant)
Review: Toolbox → Making Changes → Evaluate Performance

APPLICATION USE TARGETS

7. Motivational

Read: Foundations → Overview
Complete: Toolbox → Making Changes → Get Prepared

8. Volitional

Read: Foundations → Overview
Complete: Toolbox → Making Changes → Evaluate Performance

WEEKLY GOALS

9. Motivational

Read: Foundations → Overview
Complete: Toolbox → Making Changes → Get Prepared

10. Volitional

Read: Foundations → Overview
Complete: Toolbox → Making Changes → Evaluate Performance

11. Revise Goal

As a rule of thumb, revise goals that have not been met for two weeks in a row. Work with patient to aim for a 90% chance of success. Refer together to Toolbox, Making Changes, Develop Plan and revise goal with participant during the call.

Read: Foundations → Overview
Review: Toolbox → Making Changes → Evaluate Performance

MAINTENANCE

12. Participant Preference

As a rule of thumb, revise goals that have been met for several weeks in a row. Add something new, or make the goal more difficult. Or, if the participant desires, leave the goal as is.

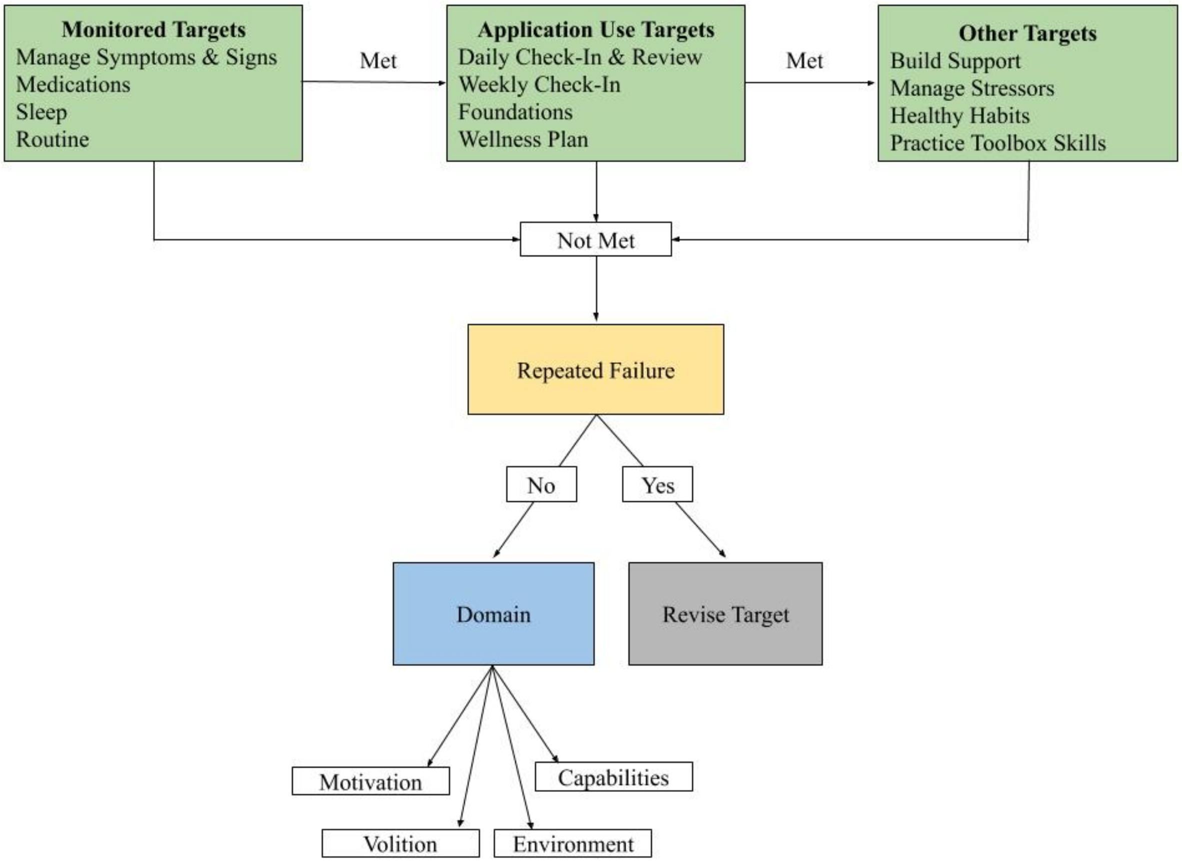
Monitored Targets
Manage Symptoms & Signs
Medications
Sleep
Routine
Met
Application Use Targets
Daily Check-In & Review
Weekly Check-In
Foundations
Wellness Plan
Met
Other Targets
Build Support
Manage Stressors
Healthy Habits
Practice Toolbox Skills
Not Met
Repeated Failure
No
Yes
Domain
Revise Target
Motivation
Capabilities
Volition
Environment

V. Ad Hoc Calls Script

LiveWell Ad Hoc Calls Script		
LWID:____________________	Coach:____________________	Date of session:____________

1. SEVERE SYMPTOMS (Crisis Wellness Rating)

Definition: Participant with daily wellness rating of +4 or -4
Get prepared with data since last call…

Medications:	Sleep:	Routine:	Wellness Ratings:	Use of App (check ins):

Daily Review feedback category:	LiveWell Clinical Status:	Weekly Survey Scores	
		PHQ8	ASRM

Hi [name]. This is [coach]. How are you doing?

I'm calling to touch base. You reported having severe symptoms today. This can be a dangerous situation. I'd like us to understand what is going on. Would that be okay?

You rated yourself as a [+4/-4]. Is that right?

____
____
____
____

Remember that it is a crisis if you are engaging in dangerous behaviors, having psychotic symptoms, or are unable to maintain daily routines. This includes thoughts of hurting yourself. In any of these cases, you should call your psychiatrist or go to the nearest emergency department.

I'd like to ask a few questions to make sure you are safe right now. Is that okay?

____
____
____

- Complete suicidality assessment protocol.

  If suicidal ideation is low or mild then complete the crisis protocol. If suicidal ideation is more severe follow the suicidal ideation protocol.

- Complete functional assessment protocol.

  If participant is experiencing suicidal ideation and/or experiencing impairment due to symptoms and psychiatrist is already aware encourage participant to reach out to psychiatrist.

Keep monitoring symptoms and be sure to reach out to your psychiatrist if you notice any major changes. Take care.

LiveWell Ad Hoc Calls Script		
LWID:____________________	Coach:____________________	Date of session:____________

2. SEVERE SYMPTOMS (Crisis elevated PHQ8 or ASRM Score)

Definition: Participant with new onset of PHQ8 ≥ 10 or new onset of ASRM ≥ 6

Get prepared with data since last call…

Medications:	Sleep:	Routine:	Wellness Ratings:	Use of App (check ins):

Daily Review feedback category:	LiveWell Clinical Status:	Weekly Survey Scores	
		PHQ8	ASRM

Hi [name]. This is [coach]. How are you doing?

I'm calling to touch base. You reported having severe symptoms today. This can be a dangerous situation. I'd like us to understand what is going on. Would that be okay?

Your most recent weekly depression/mania rating scale was high. Does that seem right?

____
____
____
____

Remember that it is a crisis if you are engaging in dangerous behaviors, having psychotic symptoms, or are unable to maintain daily routines. This includes thoughts of hurting yourself. In any of these cases, you should call your psychiatrist or go to the nearest emergency department.

I'd like to ask a few questions to make sure you are safe right now. Is that okay?

____
____
____

- Complete suicidality assessment protocol.
- Complete functional evaluation protocol.

If participant is experience suicidal ideation and/or experiencing impairment due to symptoms and psychiatrist is already aware encourage participant to reach out to psychiatrist.

Keep monitoring symptoms and be sure to reach out to your psychiatrist if you notice any major changes. Take care.

LiveWell Ad Hoc Calls Script		
LWID:____________________	Coach:____________________	Date of session:____________

3. CLINICAL STATUS DISCREPANCY

Definition: Participants' daily wellness ratings do not match their weekly lw clinical status rating and crisis and si protocols either not activated (ongoing elevated PHQ8 or ASRM) or completed with si absent, low or mild

PHQ8 ≥ 10	LiveWell Clinical Status from last 7 days of Daily Check Ins not unwell
ASRM ≥ 6	LiveWell Clinical Status from last 7 days of Daily Check Ins not unwell
PHQ8 < 10	LiveWell Clinical Status from last 7 days of Daily Check Ins unwell
ASRM < 6	LiveWell Clinical Status from last 7 days of Daily Check Ins unwell

Get prepared with data since last call…

Medications:	Sleep:	Routine:	Wellness Ratings:	Use of App (check ins):

Daily Review feedback category:	LiveWell Clinical Status:	Weekly Survey Scores	
		PHQ8	ASRM

Hi [name]. This is [coach]. How are you doing?

I'm calling to touch base. It seems like there is a difference in your daily and weekly ratings of your health and wellness. I'd like us to understand what is going on. Would that be okay?

When the daily and weekly check ins are inconsistent, we need to consider making changes in the anchors. What do you think is going on that your weekly survey said you may be [depressed, manic/ok] and your daily check ins say you are [well, prodomal, recovering/unwell]?

____
____
____
____

Would changing anchors in your Wellness Rating Scale make the application easier to use?

____
____
____
____

Ok, so we will talk again at our next scheduled appointment. I appreciate your taking such an active part in our conversation today. Remember, if any difficulties arise in the future I am always happy to help out.

4. LOW CHECK-IN ADHERENCE

Definition: Participant missed daily check in ≥ 3 times over the last 7 days or weekly check in ≥ 2 times over the past 14 days

Get prepared with data since last call…

Medications:	Sleep:	Routine:	Wellness Ratings:	Use of App (check ins):

Daily Review feedback category:	LiveWell Clinical Status:	Weekly Survey Scores	
		PHQ8	ASRM

Hi [name]. This is [coach]. How are you doing?

I'm calling to touch base. It seems like you have not been checking in regularly on the *LiveWell* application. I'd like to understand what is going on, and to see if I can help. Would that be okay?

Any problems, questions, or concerns about using the application?

____
____
____
____

What might you or we do to get past these barriers so you can benefit most from *LiveWell*?

____
____
____
____

Ok, so we will talk again at our next scheduled appointment. I appreciate your taking such an active part in our conversation today. Remember, if any difficulties arise in the future I am always happy to help out.

LiveWell Ad Hoc Calls Script		
LWID:____________________	Coach:____________________	Date of session:____________

5. POOR SENSOR DATA

Definition: Phone sensor data and/or watch data is missing or percent collected is <50 for >3 days.

Get prepared with data (% bins filled):

RobotHealth:	Location:	Telephony:	PhoneActivity:	PebbleActivity:

Hi [name]. This is [coach]. How are you doing?

I'm calling to touch base. It seems like we are not getting data from your study phone [and/or study watch]. I'd like to understand what is going on, and to see if I can help. Would that be okay?

Have you been able to carry the charged study phone at all times? If no, what's your sense of why not? [If carrying phone, troubleshoot loss of data]

____
____
____
____

If applicable: Have you been able to wear your charged study watch at all times? If no, why not? [If wearing watch, troubleshoot loss of data]

____
____
____
____

Any problems, questions, or concerns about using the study equipment?

____
____
____
____

[If technological issues ruled out]: What might you or we do to get past these barriers?

____
____
____
____

Ok, so we will talk again if we are not getting data in the future. I appreciate you talking with me today. Remember, if any difficulties arise I am always happy to help out.

VI. Suicidality Assessment Protocol

LW-SA Protocol: LiveWell Suicidality Assessment Protocol

ID:____________________ Date:__________________ Interviewer:_____________

Ask the following questions with regard to the last week or two.

A. COLUMBIA-SUICIDE SEVERITY RATING SCALE (C-SSRS)

A1. Wish to be Dead: **Have you wished you were dead or wished you could go to sleep and not wake up?** If yes, describe:

☐ Yes

☐ No

A2. Suicidal Thoughts without Method: **Have you actually had any thoughts of killing yourself?** If yes, describe:

☐ Yes

☐ No → If Wish to be Dead (A1) "No", Proceed to Action Level Low (D1).
If Wish to be Dead (A1) "Yes" Proceed to Provider Aware (C1).

A3. Suicidal Thoughts with Method: **Have you been thinking about how you might kill yourself?** If yes, describe:

☐ Yes

☐ No

A4. Suicidal Intent without Plan: **Have you had these thoughts and had some intention of acting on them?** If yes, describe:

☐ Yes → KEEP ON PHONE, Proceed to Action Level Severe (D6).

☐ No

A5. Suicidal Intent with Plan: **Have you started to work out or worked out the details of how to kill yourself? Do you intend to carry out this plan?** If yes, describe:

☐ Yes → KEEP ON PHONE, Proceed to Action Level Severe (D6).

☐ No

B. CAN CONTROL THOUGHTS/IMPULSES

B1. Can you control these thoughts or impulses and not act on them?

☐ Yes

☐ No → KEEP ON PHONE, Proceed to Action Level Severe (D6).

C. PROVIDER AWARE

C1. Have you told your psychiatrist or psychiatric nurse practitioner about these thoughts of suicide or hurting yourself?

☐ Yes

☐ No → If Suicidal Thoughts With Method (A3) "Yes" proceed to Action Level Moderate 2 (D5), else proceed to Action Level Mild 2 (D3).

C2. Have you discussed all the information you have given me today about these thoughts with your psychiatrist or psychiatric nurse practitioner?

☐ Yes → If Suicidal Thoughts With Method (A3) "Yes" proceed to Action Level Moderate 1 (D4), else proceed to Action Level Mild 1 (D2).

☐ No → If Suicidal Thoughts With Method (A3) "Yes" proceed to Action Level Moderate 2 (D5), else proceed to Action Level Mild 2 (D3)

LW-SA Protocol: LiveWell Suicidality Assessment Protocol
ID:____________________ Date:__________________ Interviewer:_____________

D. ACTION BY RISK LEVEL		
**Level**	**Action**	**Notes**
**D1. Low**<br>• No wish/No thoughts (A1 No/A2 No)	1. Inform Site PI ______________ (Initials/Date/Time)<br>2. Complete Follow-up Documentation (E)	
**D2. Mild 1**<br>• Wish/Without Thoughts (A1 Yes/A2 No)<br>OR<br>• Thoughts/Can Control (A2 Yes/B1 Yes, A3-5 No)<br>AND<br>• Provider Aware (C1+C2 Yes)	1. Inform Site PI ______________ (Initials/Date/Time)<br>2. Complete Follow-up Documentation (E)	
**D3. Mild 2**<br>• Wish/Without Thoughts (A1 Yes/A2 No)<br>OR<br>Thoughts/Can Control (A2 Yes/B1 Yes, A3-5 No)<br>AND<br>• Provider Unaware (C1or C2 No)	1. Inform Site PI ___________ (Initials/Date/Time)<br>2. Inform Primary Provider ___________ (Inform participant informing provider) (Initials/Date/Time)<br>3. Complete Follow-up Documentation (E)	
**D4. Moderate 1**<br>• Method/Can Control (A3 Yes/B1 Yes)<br>AND<br>• No Intent or Plan (A4+A5 No)<br>AND<br>• Provider Aware (C1+C2 Yes)	1. Consult Site PI _____________ (Initials/Date/Time)<br>2. Complete Follow-up Documentation (E)	
**D5. Moderate 2**<br>• Method/Can Control (A3 Yes/B1 Yes)<br>AND<br>• No Intent or Plan (A4+A5 No)<br>AND<br>• Provider Unaware (C1 or C2 No)	1. Consult Site PI _____________ (Initials/Date/Time)<br>2. Inform Primary Provider ___________ (Inform participant informing provider) (Initials/Date/Time)<br>3. Complete Follow-up Documentation (E)	
**D6. Severe**<br>• Thoughts/Cannot Control (A2 Yes/B1 No)<br>OR<br>• Method/Cannot Control (A3 Yes/B1 No)<br>OR<br>• Intent (A4 Yes)<br>OR<br>• Plan (A5 Yes)	1. Keep patient on phone<br>2. Consult Site PI ASAP _____________ (Initials/Date/Time)<br>OR Call 911 _____________ (Initials/Date/Time)<br>3. Inform Primary Provider ____________	

E. FOLLOW-UP DOCUMENTATION

	(Inform participant informing provider) (Initials/Date/Time)<br>4. Complete Follow-up Documentation (E)	

1. Follow-up recommended? ☐ No ☐ Yes

If yes, complete follow-up based on recommendations made in Action Documentation.

Was follow-up completed?

☐ No → Why not ____

☐ Yes → Who _______________________ Date ________________ Time ____________

Notes:

____

____

____

____

2. Reviewed by Study PI?

☐ No → Why not ____

☐ Yes → Date ________________ Time ____________

Notes:

____

____

____

____

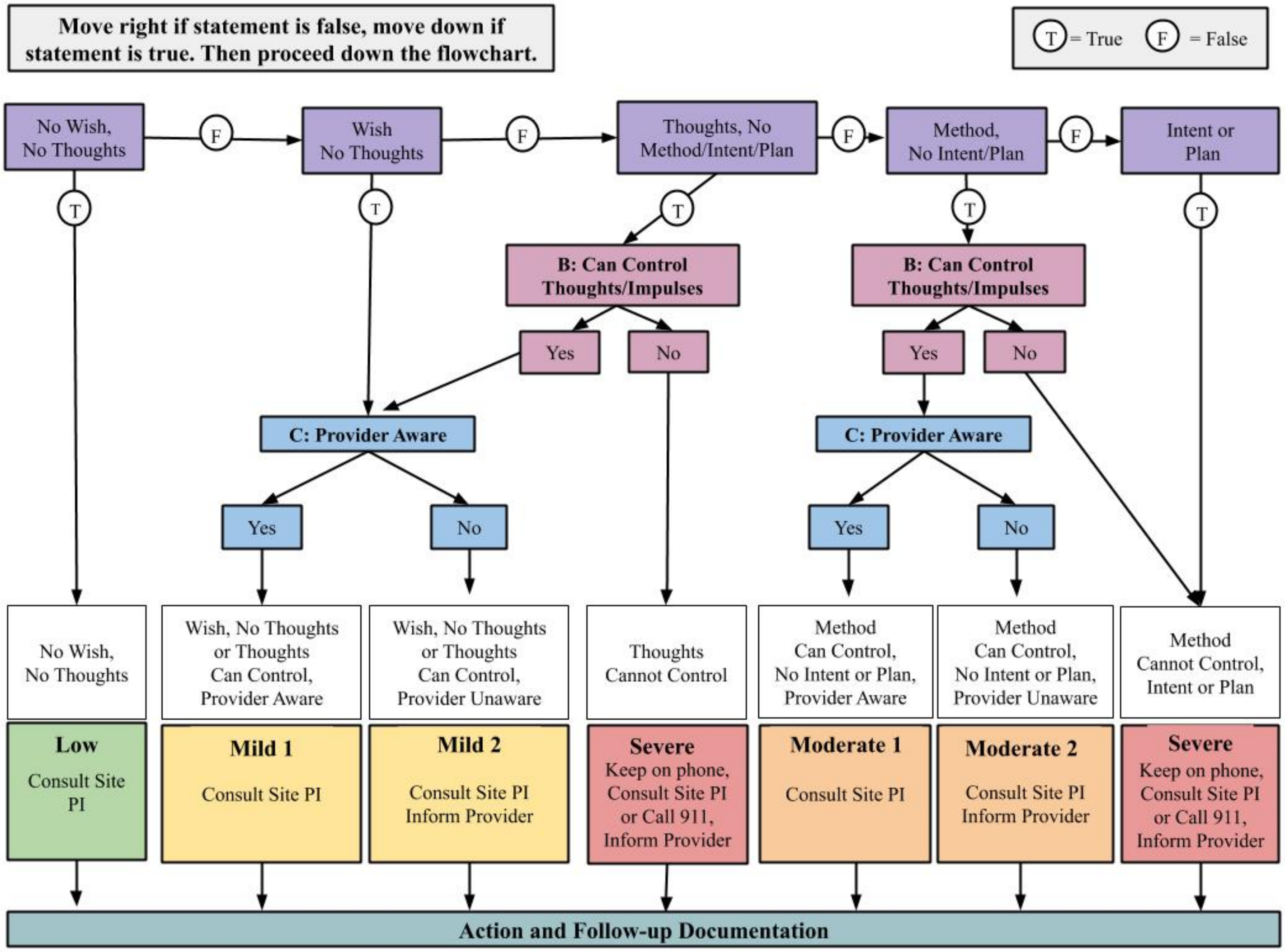
Move right if statement is false, move down if statement is true. Then proceed down the flowchart.
T = True
F = False
No Wish, No Thoughts
Wish No Thoughts
Thoughts, No Method/Intent/Plan
Method, No Intent/Plan
Intent or Plan
B: Can Control Thoughts/Impulses
Yes
No
C: Provider Aware
No Wish, No Thoughts
Wish, No Thoughts or Thoughts Can Control, Provider Aware
Wish, No Thoughts or Thoughts Can Control, Provider Unaware
Thoughts Cannot Control
Method Can Control, No Intent or Plan, Provider Aware
Method Can Control, No Intent or Plan, Provider Unaware
Method Cannot Control, Intent or Plan
Low
Consult Site PI
Mild 1
Consult Site PI
Mild 2
Consult Site PI Inform Provider
Severe
Keep on phone, Consult Site PI or Call 911, Inform Provider
Moderate 1
Consult Site PI
Moderate 2
Consult Site PI Inform Provider
Severe
Keep on phone, Consult Site PI or Call 911, Inform Provider
Action and Follow-up Documentation

Suicidal Ideation Severe Risk

Severe risk for suicide requires that we take immediate action in order to secure, as best as possible, the participant's safety.

INTRODUCING THE IDEA OF FURTHER EVALUATION…

Severe 1. Page site PI. If they do not respond within 5 minutes call 911.

It sounds like you're really struggling right now, and I'm sorry you're in so much pain. The thoughts you're having are going to pass. That said, they are serious and we need to make sure you are safe. I'd like us to consult our study clinician to get feedback about your situation. We'll stay on the phone while I do this.

Severe 2. Call 911. And then consult site PI.

It sounds like you're really struggling right now, and I'm sorry you're in so much pain. The thoughts you're having are going to pass. That said, we need to make sure you are safe until you feel better. I am going to call the paramedics and have them take you in to the emergency room for an evaluation. We'll stay on the phone while I do this. Where are you at right now?

To the 911 operator you can say something like…

Hi*, this is [name and affiliation]. I'm a research assistant for a clinical study and have a participant who is at risk for suicide. They need to be taken to the emergency room for an evaluation.*

RESPONDING TO NON-COLLABORATIVE RESPONSES…

- If they <u>change their answers</u> and/or try to convince you they are safe…

I hear what you're saying. But this is a dangerous situation and we need to err on the side of caution. Your safety and distress are what's most important.

- It would be logistically <u>problematic or embarrassing</u> (e.g., home alone with children, at work or public place)…

Try to do some problem solving to address concerns.

- If they <u>refuse to share location</u>…

First try to address any concerns or reservations that they have about going to the emergency room. Empathize, problem solve, and highlight that addressing their safety and distress is the most important.

- If they <u>hang up</u>…

Call them back and try to reengage them. If you get voicemail leave a message that you are required to call 911 and report our concerns, and that you will be contacting their psychiatrist as well.

- If they are in the <u>dangerous situation</u> (i.e., standing on bridge, driving and threatening an accident)…

Keep them engaged and talking. Try to convince them to exit the dangerous situation, for now.

VII. Suicidality Assessment Protocol Quiz

Name:
Date:

LiveWell Suicidality Assessment Protocol Quiz

Protocol Structure

1. At what point do you consult the Site PI following completion of an SI Protocol?
    a. You should always consult the Site PI
    b. Only when the participant provides new information that his/her psychiatrist does not know about
    c. Only when the participant's risk level is Severe
    d. Only when the participant has thoughts of killing himself/herself with method
2. In which of the following circumstances would you inform the participant's psychiatrist about thoughts of suicide?
    a. Any time the participant has thoughts of killing himself/herself, regardless of severity level
    b. When the participant has thoughts of killing himself/herself with a method, regardless of whether his/her psychiatrist is aware
    c. When the participant has thoughts of wanting to die that his/her psychiatrist is aware of
    d. When the participant provides new information regarding thoughts of killing himself/herself that his/her psychiatrist is not aware of
3. When should you keep the participant on the phone and call/page the Site PI immediately?
    a. If in the past 2 weeks the participant has had thoughts of killing himself/herself with a method, but no intent or plan
    b. If in the past 2 weeks the participant has had thoughts of killing himself/herself with a method, some intention of acting on these thoughts and has begun to work out details of how to kill himself/herself
    c. If the participant has had thoughts of killing himself/herself with method and feels he/she cannot control those thoughts
    d. Both B & C

Scenarios *Characterize the following examples of suicidal ideation according to wishes, thoughts, method, intent and plan:*

4. "I had moments in the past week when I wanted to end things and be gone…I thought about overdosing, but I wouldn't actually go through with it."
    a. Thoughts of killing himself/herself with method, plan, and intent
    b. Thoughts of killing himself/herself without method, plan, or intent
    c. Thoughts of killing himself/herself with method and plan but no intent
    d. Thoughts of killing himself/herself with method but no plan or intent
5. "I thought about hanging myself about a week ago. I bought a rope from the store and figured out when my mom would be out of the house. I wanted to end things right then, but I chickened out."
    a. Thoughts of killing himself/herself with method, plan, and intent
    b. Thoughts of killing himself/herself without method, plan, or intent.
    c. Thoughts of killing himself/herself with method and plan but no intent
    d. Thoughts of killing himself/herself with method but no plan or intent
6. "I've been pretty depressed over the past 2 weeks. I've been wishing I would just not wake up one morning, so I wouldn't have to face the next day…I've been down but haven't thought about killing myself."
    a. Thoughts of wanting to die
    b. Thoughts of killing himself/herself without method
    c. Thoughts of killing himself/herself with method
    d. Both A & B

Employee Sign Off (Once Trainee has received score of 100%)
Name: ____________________________________ Date: ________________

Name:
Date:

LiveWell Suicidality Assessment Protocol Quiz

Protocol Structure

1. At what point do you consult the Site PI following completion of an SI Protocol?
    a. You should always consult the Site PI
    b. Only when the participant provides new information that his/her psychiatrist does not know about
    c. Only when the participant's risk level is Severe
    d. Only when the participant has thoughts of killing himself/herself with method
2. In which of the following circumstances would you inform the participant's psychiatrist about thoughts of suicide?
    a. Any time the participant has thoughts of killing himself/herself, regardless of severity level
    b. When the participant has thoughts of killing himself/herself with a method, regardless of whether his/her psychiatrist is aware
    c. When the participant has thoughts of wanting to die that his/her psychiatrist is aware of
    d. When the participant provides new information regarding thoughts of killing himself/herself that his/her psychiatrist is not aware of
3. When should you keep the participant on the phone and call/page the Site PI immediately?
    a. If in the past 2 weeks the participant has had thoughts of killing himself/herself with a method, but no intent or plan
    b. If in the past 2 weeks the participant has had thoughts of killing himself/herself with a method, some intention of acting on these thoughts and has begun to work out details of how to kill himself/herself
    c. If the participant has had thoughts of killing himself/herself with method and feels he/she cannot control those thoughts
    d. Both B & C

Scenarios *Characterize the following examples of suicidal ideation according to wishes, thoughts, method, intent and plan:*

4. "I had moments in the past week when I wanted to end things and be gone…I thought about overdosing, but I wouldn't actually go through with it."
    a. Thoughts of killing himself/herself with method, plan, and intent
    b. Thoughts of killing himself/herself without method, plan, or intent
    c. Thoughts of killing himself/herself with method and plan but no intent
    d. Thoughts of killing himself/herself with method but no plan or intent
5. "I thought about hanging myself about a week ago. I bought a rope from the store and figured out when my mom would be out of the house. I wanted to end things right then, but I chickened out."
    a. Thoughts of killing himself/herself with method, plan, and intent
    b. Thoughts of killing himself/herself without method, plan, or intent.
    c. Thoughts of killing himself/herself with method and plan but no intent
    d. Thoughts of killing himself/herself with method but no plan or intent
6. "I've been pretty depressed over the past 2 weeks. I've been wishing I would just not wake up one morning, so I wouldn't have to face the next day…I've been down but haven't thought about killing myself."
    a. Thoughts of wanting to die
    b. Thoughts of killing himself/herself without method
    c. Thoughts of killing himself/herself with method
    d. Both A & B

Employee Sign Off (Once Trainee has received score of 100%)
Name: ______________________________________ Date: ________________

VIII. Functional Evaluation Protocol

LW-IE: LiveWell Functional Evaluation Protocol

ID:_____________ Date:_____________ Interviewer:_____________

Ask the following questions with regard to the last week or two.

A. CHANGES IN FUNCTIONING

1. Self-Care: Have there been any changes in your sleep, eating, or hygiene and grooming?
If so, describe:

☐ Yes
☐ No

2. Social Behavior: Have there been any changes in your interactions with other such as withdrawal, associating with strangers, or conflicts?
If so, describe:

☐ Yes
☐ No

3. Impulse Control: Have there been any changes in your sexual activity, spending habits, or substance use?
If so, describe:

☐ Yes
☐ No

4. Family Obligations: Are you having any problems keeping up with family responsibilities?
If so, describe:

☐ Yes
☐ No

5. Work Obligations: Are you having any problems keeping up with work, school, or volunteer responsibilities?
If so, describe:

☐ Yes → PROCEED TO NEW INFORMATION (SECTION B)
☐ No → IF ANY OF A1-A4 ARE "YES", PROCEED TO NEW INFORMATION (SECTION B)
IF ALL OF A1-A5 ARE "NO", PROCEED TO ACTION LEVEL C1 (ABSENT)

B. NEW INFORMATION

1. Have you told your psychiatrist or psychiatric nurse practitioner about these changes or problems?

☐ Yes
☐ No → PROCEED TO ACTION LEVEL C3 (PRESENT 2)

2. Have you discussed all the information you have given me today about these changes or problems with your psychiatrist or psychiatric nurse practitioner?

☐ Yes → IF QUESTION 1 "YES", PROCEED TO ACTION LEVEL C2 (PRESENT 1)
☐ No → PROCEED TO ACTION LEVEL C3 (PRESENT 2)

C. ACTION BY RISK LEVEL

Level	Action	Notes
**C1. Absent**<br>• No changes in functioning endorsed	1. Informed Site PI ____________ (Initials/Date/Time)<br>2. Complete Follow-up Documentation (D)	
**C2. Present 1**<br>• Any changes in functioning endorsed<br>• Provider Aware	1. Informed Site PI ____________ (Initials/Date/Time)<br>2. Complete Follow-up Documentation (D)	
**C3. Present 2**<br>• Any changes in functioning endorsed<br>• Provider Unaware	1. Consult Site PI ____________ (Initials/Date/Time)<br>2. Per Site PI, Inform Primary Provider? Y / N<br>3. If Yes, Inform Primary Provider ____________ (Inform participant informing provider) (Initials/Date/Time)<br>4. Complete Follow-up Documentation (D)	

D. FOLLOW-UP DOCUMENTATION

1. Follow-up recommended? ☐ No ☐ Yes
If yes, complete follow-up based on recommendations made in Action Documentation.

Was follow-up completed?

☐ No → Why not ____

☐ Yes → Who ____________________ Date ______________ Time ___________
Notes:

____

____

____

____

2. Reviewed by Study PI?

☐ No → Why not ____

☐ Yes → Date ______________ Time ___________
Notes:

ID:______________ Date:______________ Interviewer:______________

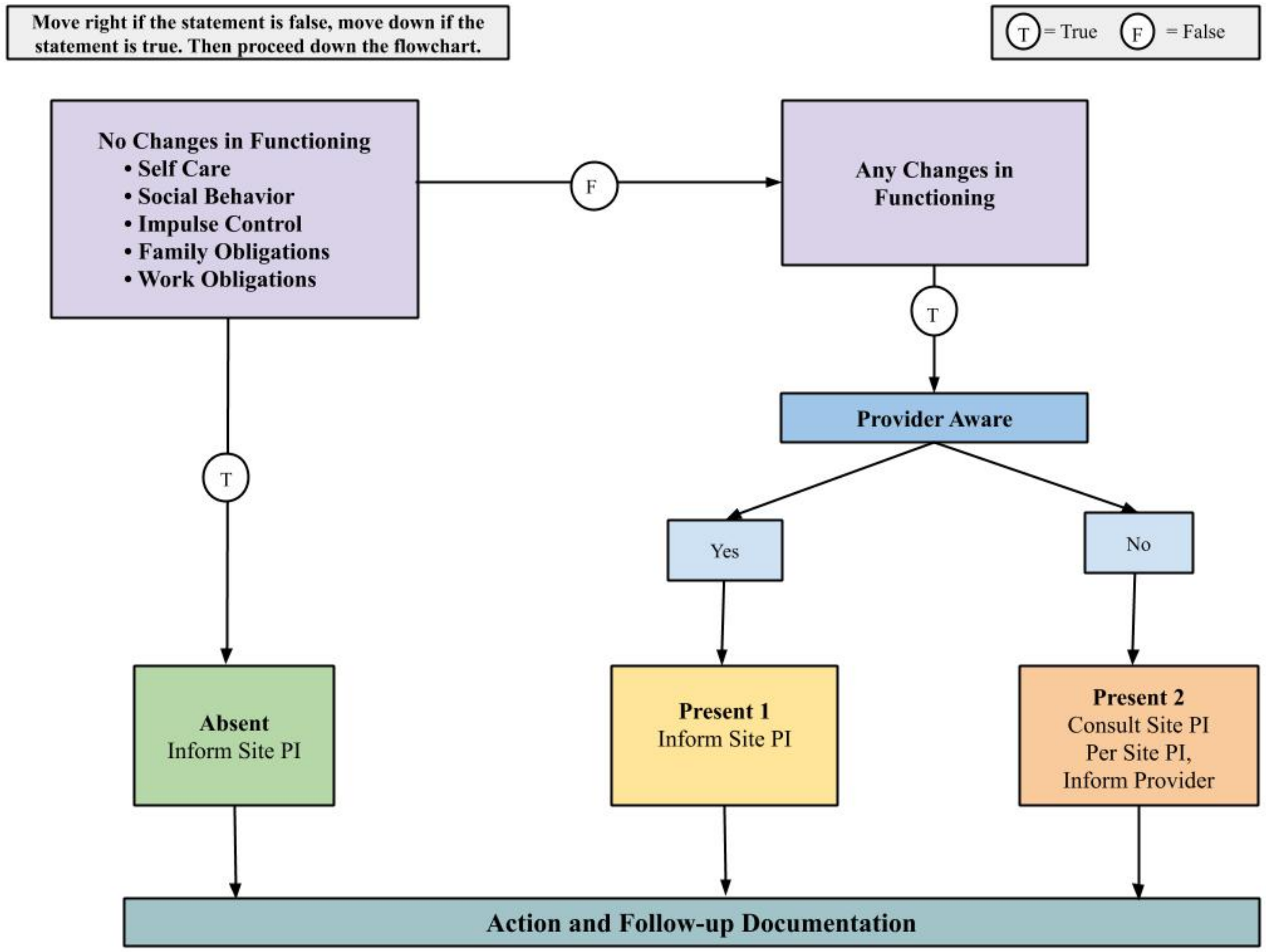

IX. Fidelity Rating Scale

LW-CFRS: LiveWell Coach Fidelity Rating Scale V1
LWID:_______ Date:________ Follow-Up Month: ____________ Coach:__________ Administer:__________

Directions: Assess the coach on a scale of 0 to 2 for each item. If the descriptions for any given item occasionally do not appear to apply to the session being rated, then record a more general impression. Do not leave any item blank. For all items, focus on the coach's skill, taking into account the challenges presented by the patient.

0 = Not at all, 1 = Needs Improvement, 2 = Satisfactory

Coach:____________________________ Patient:___________________________ Date of session:_____________________
Rater:_____________________________ Modality:__________________________ Date of rating:______________________

I. AGENDA SETTING AND PERMISSION SEEKING

1. The coach sets an agenda in a way that invites patient to talk about behavior change.	0 1 2
2. The coach demonstrates sensitivity to talking about other issues.	0 1 2

II. PATIENT ACCOUNTABILITY

3. The coach facilitates goal setting.	0 1 2
4. The coach reviews use of application.	0 1 2
5. The coach reviews goal achievement.	0 1 2

III. THE WHY AND HOW OF BEHAVIOR CHANGE

6. The coach elicits how the patient thinks and feels about using the application.	0 1 2
7. The coach encourages patient to talk about current behavior or status quo.	0 1 2
8. The coach encourages patient to talk about behavior change.	0 1 2
9. The coach asks questions to elicit how patient thinks and feels about behavior change.	0 1 2
10. The coach uses active listening statements when patient talks about behavior change.	0 1 2

VI. THE WHOLE CONSULTATION

11. The coach acknowledges challenges about behavior change that the patient faces.	0 1 2
12. When the coach provides information, it is sensitive to patient concerns and understanding.	0 1 2
13. The coach actively conveys respect for patient choice about behavior change.	0 1 2
14. Coach and patient *exchange* ideas about *how* patient could change current behavior.	0 1 2

V. *INITIAL SESSION ONLY*

15. The coach obtains information about the patient's reasons for participation.	0 1 2
16. The coach obtains information about the patient's expectations for the program.	0 1 2
17. The coach provides patient with information about the application and bipolar disorder.	0 1 2
18. The coach works with the patient to establish the coach's role.	0 1 2
19. The coach works with the patient to establish how coach and patient will work together.	0 1 2
20. The coach obtains information about the patient's challenges towards participation.	0 1 2
21. The coach is able to elicit enthusiasm and commitment talk from the patient.	0 1 2

Coding Schema

DOMAIN I: AGENDA SETTING AND PERMISSION SEEKING

1. The coach sets an agenda in a way that invites the patient to talk about behavior change.

A high score: The coach explicitly asks the patient's permission to talk about behavior change, making it clear that the patient is not obliged to make any decisions regarding their behavior.

Example: "Before we start, I'd just like to make it clear that I am not here to tell you what to do or to force you to make decisions you don't feel ready to make. I am here to understand you. We don't have to talk about anything you don't want to talk about. Now I understand you are here to talk (agenda items). Would it be okay with you if we had a chat about that now?"

A low score: The coach fails to ask the patient about a willingness to talk about behavior change and does not give them an opportunity to speak, giving the impression that the patient has little choice in the matter!

2. The coach demonstrates sensitivity to talking about other issues.

An issue can be anything of concern to the patient, whether it is connected to the behavior in question or not.

A high score: The patient is given choice in what to talk about, because, for example, the coach goes through an agenda setting process in which the patient is encouraged to talk about other health behaviors, or other issues not immediately connected to behavior change.

Example: "So, since our last discussion you decided to make some changes in your sleep schedule. That must have been a challenge. I know we're going to focus on that in part, but I'm wondering if there is anything else concerning you that you would like to talk about today as well."

A low score: The coach does not give the patient any choice about what to talk about, and proceeds with the consultation discussing in turn what s/he feels are the most important issues.

DOMAIN II: PATIENT ACCOUNTABILITY

3. The coach facilitates goal setting.

Coach and patient exchange ideas about how the patient could change current behavior.

A high score: The coach actively encourages the patient to brainstorm a number of strategies that may help them change their behavior. With encouragement, the patient offers the most ideas, and the coach also makes suggestions. Emphasis is on <u>process</u> rather than outcomes, but process clearly linked to outcomes.

A low score: The coach does not encourage the patient to brainstorm. There is no exchange about a range of possibilities. The patient does not suggest any. Instead, it is the coach only who suggests ideas for change.

4. The coach reviews use of application.

A high score: Elicits information about how the patient thinks and feels about using the application.

A low score: Does not ask patient about their experiences with the application.

5. **The coach reviews goal achievement.**

   A high score: The coach actively engages the patient in a discussion about the extent to which they met their goals. If patient reached process goal, coach explores link to outcome goals. If patient did not reach process goal, coach focuses on lessons learned.

   A low score: Coach does not discuss goal achievement, or if they did, did not link process (effort) to outcome.

DOMAIN III: THE WHY AND HOW OF BEHAVIOR CHANGE

6. **The coach elicits how the patient thinks and feels about using the application.**

   A high score: The coach uses a range of (mainly open-ended) questions to draw as much information from the patient as possible about their thoughts and feelings about using the application.

   A low score: The coach does not ask the patient any questions regarding their thoughts and feelings about using the application, or asks only closed-ended questions that do not give the patient the opportunity to express their thoughts and feelings about using the application.

7. **Coach encourages patient to talk about current behavior or status quo.**

   A high score: The coach encourages the patient to talk freely about what they both like and/or dislike about their current behavior/status quo. They may do this in a variety of ways, for example through using active listening skills, to gain an understanding of the patient’s perspective.

   A low score: The coach does not actively encourage the patient to talk about what they like and/or dislike about their current behavior/status quo.

8. **Coach encourages patient to talk about behavior change.**

   A high score: The coach encourages the patient to talk freely about what they feel the positive and negative aspects of behavior change would be for them. They may do this in a variety of ways, for example through using active listening, to gain an understanding of the patient’s perspective.

   A low score: The coach does not actively encourage the patient to talk about what they feel the positive and negative aspects of behavior change would be for them.

9. **Coach asks questions to elicit how patient thinks and feels about behavior change.**

   A high score: The coach uses a range of (mainly open-ended) questions to draw as much information from the patient as possible about their thoughts and feelings towards the topic of behavior change.

   A low score: The coach does not ask the patient any questions regarding their thoughts and feelings about behavior change, or asks only closed-ended questions that do not give the patient the opportunity to express their thoughts and feelings about the topic of behavior change.

10. **Coach uses active listening when patient talks about behavior change.**

    A high score: The coach uses a range of active listening skills while the patient is talking about behavior change to clarify whether s/he has understood what the patient has said, and/or to encourage the patient to amplify further. Active listening includes open-ended questions, affirmations, reflections, and summary statements (OARS).

A low score: The coach makes no active listening statements when the patient is talking about behavior change.

DOMAIN VI: THE WHOLE CONSULTATION

11. Coach acknowledges challenges about behavior change that the patient faces.

A high score: The coach regularly and explicitly acknowledges the challenges that may be facing the patient. This affirmation is done by focusing on the personal strengths that the patient has in the face of these challenges.

Example: "Goodness, I can see why it is so hard for you to change. You have so much stress to deal with in your life right now. It must be really hard. But even though this is really difficult, you have already begun to make changes to your lifestyle following your recent manic episode, and you've managed to keep them up even though it's tough."

A low score: The coach does not make any explicit acknowledgment the challenges that face the patient, and does not focus on any of the personal strengths that the patient has.

12. When coach provides information, it is sensitive to patient concerns and understanding.

A high score: The coach tries to understand what the patient knows and wants to know, and also elicits their personal reaction to information provided.

A low score: The coach gives information to the patient without asking whether the patient wants or needs information. The personal relevance of the information is not drawn out of the patient, but provided by the coach. A low score would also be given if the patient requests information from the coach, and the coach does not provide any.

13. Coach actively conveys respect for patient choice about behavior change.

A high score: The coach openly acknowledges and accepts patient choice even if this does not fit in with coach's agenda. The coach does not put any pressure on the patient to change their behavior.

Example: 'At the end of the day, it's your choice. It's up to you if you want to make any changes, and it's about what is realistic and manageable for you. You don't have to do anything you don't want to do.'

A low score: The coach does not acknowledge or accept patient choice.

14. Coach and patient *exchange* ideas about the *how* patient could change current behavior.

A high score: The coach actively encourages the patient to brainstorm a number of strategies that may help them change their behavior. With encouragement, the patient offers the most ideas, and the coach also makes suggestions.

A low score: The coach does not encourage the patient to brainstorm. There is no exchange about a range of possibilities. The patient does not suggest any ideas. Instead, it is the coach only who suggests ideas for change.

V. *INITIAL SESSION ONLY*

For items 15 through 21, mark a high score if the coach covered the topic area in a way that was comprehensive and sensitive to the patient's needs. Mark a low score if the coverage was either inadequate and/or did not take into account that patient's seeming understanding or readiness.
